# Gender, Age, Hunger, and Body Mass Index as Factors Influencing Portion Size Estimation and Ideal Portion Sizes

**DOI:** 10.3389/fpsyg.2022.873835

**Published:** 2022-05-11

**Authors:** Kalina Duszka, Markus Hechenberger, Irene Dolak, Deni Kobiljak, Jürgen König

**Affiliations:** Department of Nutritional Sciences, University of Vienna, Vienna, Austria

**Keywords:** portion size, eating behavior, portion estimation, nutrition, food choices

## Abstract

Portion sizes of meals have been becoming progressively larger which contributes to the onset of obesity. So far, little research has been done on the influence of body weight on portion size preferences. Therefore, we assessed whether Body Mass Index (BMI), as well as other selected factors, contribute to the estimation of food portions weight and the subjective perception of portion sizes. Through online questionnaires, the participants were asked to estimate the weight of pictured foods in the first study. In the second study, the participants indicated how the depicted varying portion sizes of different meals relate to their actual consumed real-life portion sizes. A total of 725 and 436 individuals were included in the statistical analysis in the first and second study, respectively. BMI and gender had a small effect on the capacity to estimate the weight of foods. The main predictor for portion size choices was the factor gender with men estimating ideal portion sizes as larger than women. Further, age and hunger together with external and restrictive eating behaviors were among the deciding factors for portion size choices. As expected, externally motivated eaters chose bigger portions while restrictive individual smaller ones. Gender- and age-related differences in portion size preferences likely reflect distinct energy requirements. The individuals with a higher BMI do not differ strongly from other BMI groups in their portion-related preferences. Therefore, other factors such as meal frequency, snacking, or a lifestyle, may contribute more to the onset, development, and maintenance of overweight.

## Introduction

It has been repeatedly stated that the prevalence of overweight subjects was steadily increasing over the past decades. Among adults worldwide, 39% are of BMI indicating overweight and 13% with obesity, with obesity nearly tripling from 1975 to 2016 (WHO, 2018). Reasons for this increasing prevalence are varied, ranging from intrinsic factors affecting energy balance to psychological factors influencing eating behavior. Among these and other factors, consumers in many regions of the world are constantly being confronted with increasing serving sizes (Livingstone and Pourshahidi, 2014; Benton, 2015). Already in 2003 portion sizes of ready-to-eat prepared foods exceeded the standard portion sizes used by the USDA Food Guide Pyramid or the FDA label by at least a factor of 2 (bagels, sodas) and sometimes as much as 8 (cookies) (Young and Nestle, 2003). The effect of perceiving portion sizes much larger than physiologically required has become known as portion distortion (Schwartz and Byrd-Bredbenner, 2006). A portion is defined as the amount of food or dish that is consumed by an individual during a meal (Hackett and The, 2009). While portion defines the amount of food that a person actually consumes, serving is the term used to define the recommended portion size suggested by experts and food producers. Consequently, portion size can reflect numerous perceptions depending on the specific evaluator: a consumer, a restaurant owner, a food producer, or a nutritional association (Lewis et al., 2012). In reality, these different concepts of portion and sizes are often divergent. The relationship of increasing rates of overweight population in regard to portion size is the subject of a few studies, especially with respect to the Portion-Size-Effect (Young and Nestle, 2012; Livingstone and Pourshahidi, 2014; O’Brien et al., 2015). Portion-Size-Effect indicates that individuals will eat more when they are served a larger portion of food making a connection between portion size and energy intake (Benton, 2015; English et al., 2015; Steenhuis and Poelman, 2017). This effect is consistent across all BMI levels (Rolls et al., 2002; Marchiori et al., 2011). Conscious as well as subconscious factors or mechanisms responsible for influencing the Portion-Size-Effect include unit bias (Geier et al., 2006), expected satiety (Brunstrom and Rogers, 2009), energy compensation (Benton, 2015), bite-size (Almiron-Roig et al., 2018), visual stimuli (Marchiori et al., 2011; Penaforte et al., 2014), financial aspects (Benton, 2015; Steenhuis and Poelman, 2017), and portion norms (Lewis et al., 2015). In the context of Portion-Size-Effect, the subconscious mechanisms are reflected in the lack of differentiation when people eat from a large portion versus a small portion, despite consuming more (Levitsky and Youn, 2004) even after being informed about the difference in portions sizes (Spanos et al., 2015; Reily et al., 2016). Furthermore, consumption of bigger portion sizes and lack of restriction was observed even when the portions are served consistently over multiple subsequent meals (Rolls et al., 2007). Concerning portions norms, the term norm is generally seen as socially acceptable and expected behavior in specific situations. Therefore, norms influence human behavior and play an important role in the evaluation of portion sizes (Lewis et al., 2015). A distinction can be made between personal and social norms (Lewis et al., 2015).

The most accurate way of determining the amount of food consumed is through weighing meals. As this method requires a high level of compliance, it is mainly used in small sample sizes (Almiron-Roig et al., 2018). For bigger samples—methods that rely on one’s memory—such as the so-called Food Frequency Questionnaire (FFQ) and the 24-h Recall, are often implemented (Almiron-Roig et al., 2018). When applying these methods portion size estimation aids (PSEA) are frequently used as assistance (Subar et al., 2015; Faulkner et al., 2016). However, the validity of the data acquired through these methods comes with uncertainty. On the one hand, inaccurate weighing of food, as well as a change in eating habits during the survey, can lead to erroneous data. On the other hand, questionnaires have limitations, in that participants can make vague statements or have false recollections (Subar et al., 2015). Providing accurate estimates of the foods consumed is therefore important to reduce uncertainty in dietary surveys.

Despite recommendations from various nutritional societies for a balanced diet, the increasing prevalence of overweight and obesity shows that consumers are having difficulty adhering to these recommendations. A different perception of portion sizes based on individual preferences might be a reason for not adhering to dietary guidelines. Overall, the question arises of how well the consumer can estimate the amount of food when asked to report the quantity of foods consumed as multiples of portion size and whether there are differences between overweight or obese and normal weight in the portion size assessment of different foods. On one hand, individuals with BMI over 25 tend to be less responsive to larger food portions (Zlatevska et al., 2014); however, they are more likely to choose larger portions fueling further weight gain (Westerterp-Plantenga et al., 1996; Flegal and Troiano, 2000; Young and Nestle, 2003, 2012). Accordingly, higher BMI is associated with selecting larger amounts of food in a setup where self-selection of portion size was permitted (Burger et al., 2007; Lewis et al., 2015). However, other reports with a similar design found no difference as a function of BMI (Brunstrom et al., 2008; Embling et al., 2021). In studies allowing the choice between fixed portion sizes, some indicated that participants with obesity order larger portions than non-obese (Dodd et al., 1976) which was not confirmed by other reports (Reily et al., 2016). Thus, the issue of the relation between BMI and portion size is controversial and remains unresolved. Intuitively, the association between BMI and portion size is expected; however, it may be difficult to provide evidence by the approaches applied so far. Therefore, verification of new tactics, as well as challenging and optimization of existing tools, is needed to distinguish between the true results or merely the impact of methodology.

In this study, we studied interconnection between two aspects connected with portion size: recognition and preference. We verified the ability of participants to estimate the weight of food provided by food pictures used in photo books for nutritional surveys. We hypothesize that people with obesity and overweight show a distorted perception of food weight. In addition, we tested the subjective estimation of portion size of different foods as part of a whole meal and hypothesize that individuals with BMI in the obese and overweight range consider larger portions as their normal portion. We use an online survey as a medium allowing reaching a high number of participants of various backgrounds including age and BMIs range.

## Methods

### Study I

The survey was carried out as a cross-sectional study in the form of an online questionnaire. In order to recruit the participants, the questionnaire link was made available in internet forums and online platforms. Participation in the study was voluntary and anonymous. The questionnaire was created using the SoSciSurvey platform.^1^ In a sociodemographic questionnaire, the participants specified their height, weight, age, gender as well as their current feeling of hunger and satiety. The participants were classified into four BMI [BMI = body mass (in kg)/height2 (in m^2^)] groups: underweight (<18.5 kg/m^2^), normal weight (BMI 18.5–25 kg/m^2^), with overweight (BMI ≥ 25 kg/m^2^ to < 30 kg/m^2^), and with obesity (BMI ≥ 30 kg/m^2^). Based on their age the participants were grouped in the following categories: 16–25, 25–35, 35–45, 45–55, >55 years. Concerning hunger, the following categories were used: full, somewhat hungry, moderately hungry, and very hungry. The participants were asked to estimate in grams the weight of the presented portions of 14 foods by entering the estimated weight into an input box. The detailed description of the study design is available in Supplementary Material.

### Study II

Similarly as for study I, the data for study II were collected through an online questionnaire, using the SoSciSurvey platform and the participation was voluntary and anonymous. Within the questionnaire sociodemographic factors (gender and age) as well as personal and physiological attributes (height, weight, feelings of hunger during participation, and eating behavior) were assessed. The participants were divided into the same groups as in study I for gender, BMI, age, and hunger rating. Additionally, assessment of eating behavior has been performed using the Dutch Eating Behavior Questionnaire (DEBQ). A set of 11 meals has been prepared and varying portions sizes of each food have been photographed. For each meal, the portion sizes were presented randomly (i.e., not arranged according to size) during the questionnaire. The participants were asked to rate the portions sizes of each meal in relation to their preferred portion size during a typical eating occasion of a meal using a 100 mm-visual analog scale (VAS). More details concerning the study design are available in Supplementary Material.

### Statistical Analysis

We collected these data hypothesizing that BMI impacts recognition and choices of food portions. The collected data was stratified into corresponding groups according to gender, age, BMI, and hunger rating. The statistical analysis was performed using IBM SPSS Statistics 26.0. To test for potential differences between variables of different categories the chi-square test was used. A comparison of mean value between two groups was carried out using the Students’ *t*-test, whereas comparisons of more than two groups were made using ANOVA. Bonferroni correction for multiple testing for independent samples set the *p*-value threshold to 0.0036 for study I and 0.0045 for study II. For those factors, where interaction effects were found, an analysis of covariance with BMI as fixed factor and age, gender, and hunger rating as covariates was performed. The Kolmogorov-Smirnov test was used to determine the normal distribution of the data. Due to the partly unequal distributions, a test to check the homogeneity of the variances (Levene-Test) was carried out in conjunction with the ANOVA. If the results of the Levene test suggested an inhomogeneous distribution of the variances, a corrected, robust F-statistic (Welch-F) was applied. In the case of significantly different ANOVA results, a GT-2 Hochberg *post hoc* test was used to determine which groups differ from each other. For testing the relationship between different DEBQ categories and ideal portion size a partial correlation analysis was performed with hunger, gender, age, and BMI as control variables. The significance level was set at α = 0.05. The internal consistency of the subscales for DEBQ scores was evaluated using Cronbach’s Alpha.

### Ethics

The studies were performed in compliance with the ethical standards of the Declaration of Helsinki. Studies design was approved by the ethics committee of the University of Vienna (approval ID 00576). All participants gave their written consent to participate in the studies.

## Results

### Study I

In total 438 questionnaires were completed and screened for the occurrence of unrealistic values in terms of BMI (BMI values > 50 kg/m^2^ and < 16 kg/m^2^), age (16–100) as well as values of weight estimates of the portions (Supplementary Figure 1). If more than one unrealistic value of the 14 servings was specified, the data record was removed. Unrealistic value was defined as over ten times difference compared to the effective weight of the item. However, an exception was made for chips and butter for which all of the values were included as these items were characterized by very frequent high estimation inaccuracy indicating commonly occurring difficulties in weight estimation of high-calorie foods. A total of 436 individuals completed the questionnaire correctly and were therefore included in the statistical analysis. There were a total of 228 female (52.29%) and 208 male (47.71%) participants (Supplementary Table 1). The mean BMI of the study population is 25.26 ± 6 kg/m^2^ (Supplementary Table 1). For the statistical analysis subjects are classified into underweight, normal weight, with overweight, and with obesity accounting for 9.40, 46.33, 26.61, and 17.66% of the sample size, respectively. BMI of males (23.52 ± 5 kg/m^2^) was significantly lower than that of females (26.84 ± 6 kg/m^2^; *p* < 0.01). The subjects’ age ranged from 16 to 70 years and the average age was 33.96 ± 11 years (Supplementary Table 1). There was no difference in the age of the male and female participants (*p* = 0.75). The majority of the subjects reported feeling full (*N* = 193, 44.27%). Less of them were somewhat hungry (*N* = 62, 14.22%), moderately hungry (*N* = 108, 24.77%) or very hungry (*N* = 73, 16.74%) (Supplementary Table 1). The hunger level significantly differed between genders (*p* < 0.001) but not between BMI categories (*F* = 0.262; *p* = 0.853).

In general, the deviation from the actual values in the estimation of the portion sizes was very heterogeneous (Table 1). The weights of the foods potato chips, cake, beans, noodles, rice, pork meat, and butter were generally overestimated. High-calorie food items, chips and butter were characterized by a very strong variation in the weight estimation. On the other hand, apple compote, and berries were generally the most underestimated while potatoes, paprika, ham, and fish filet showed relatively accurate (<10% difference compared to the effective weight) estimates, but with high standard deviations.

**TABLE 1:** Results of study I for gender and BMI categories.

	Total		Gender			BMI					
Item	Real weight (g)	Mean weight estimation (g)	Females (%)	Males (%)	*p*	18.5 (%)	18.5–25 (%)	25–30 (%)	>30 (%)	*F*	*p*
Potatoes	141	146.20 ± 65	5	2	0.577	–16	4	7	7	4.527	0.004
Potato chips	20	59.52 ± 47	177	188	0.610	183	186	188	161	0.262	0.853
Cake	137	177.46 ± 70	31	23	0.127	11	28	29	29	2.905	0.035
Beans	99	170.99 ± 71	74	70	0.571	47	75	68	81	0.477	0.698
Paprika	83	93.50 ± 48	13	9	0.527	0	6	10	29	3.461	0.016
Apple compote	231	115.29 ± 58	–48	–53	0.053	–58	–50	–49	–49	1.647	0.178
Berry mix	161	125.45 ± 61	–19	–30	0.005	–31	–25	–23	–20	0.571	0.634
Noodles	109	217.96 ± 84	103	89	0.071	88	97	100	93	1.065	0.364
Rice	60	102.40 ± 49	74	67	0.361	72	71	71	68	0.332	0.802
Pork meat	111	185.02 ± 79	72	56	0.021	49	67	59	72	1.542	0.203
Ham	49	43.95 ± 33	–13	–8	0.428	20	–12	–15	–18	0.208	0.891
Fish filet	97	101.33 ± 44	9	0	0.044	–8	5	2	13	2.428	0.065
Butter	10	19.32 ± 21	94	122	0.338	119	131	76	85	0.955	0.414
Scrambled eggs	100	87.78 ± 49	–8	–16	0.110	–29	–11	–11	–7	1.995	0.114

*The results were calculated as a percentage of over or under estimation of the weight of each product.*

**FIGURE 1:**
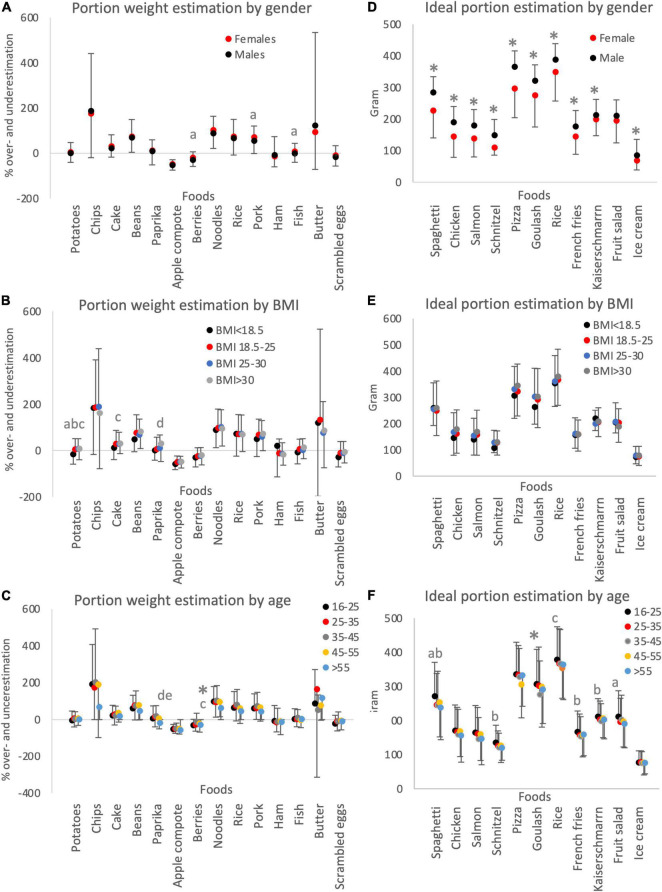
The impact of the main factors affecting portion estimation. The data collected for portion weight estimation **(A–C)** and ideal portion estimation **(D–F)** were analyzed for subgroups identified based on gender **(A,D)**, BMI **(B,E)**, and age **(C,F)**. The statistical difference in **(A,D)** were determined by Students’ *t*-test for independent samples with a *p*-value threshold accounting for multiple testing for study I *p* < 0.0036 and for study II *p* < 0.045. In **(A)**, *p* < 0.05 was indicated with a. One-way ANOVA was performed to compare groups in **(B,C,E,F)**. *Indicates statistical significance for **(C,D,F)**. In **(B)**, a indicates *p* < 0.05 between groups BMI < 18.5 and BMI 18.5–25; b indicates *p* < 0.05 between groups BMI < 18.5 and BMI 25–30; c indicates *p* < 0.05 between groups BMI < 18.5 and BMI > 30; d indicates *p* < 0.05 between groups BMI 18.5–25 and BMI > 30. In **(C,F)**, *indicates statistical significance between age groups 16–25 and 35–45; a indicates *p* < 0.05 between age groups 16–25 and 25–35; b indicates *p* < 0.05 between age groups 16–25 and 35–45; c indicates *p* < 0.05 between age groups 16–25 and 45–55; d indicates *p* < 0.05 between age groups 25–30 and > 55; e indicates *p* < 0.05 between age groups 35–45 and > 55. The data are presented as mean ± SD.

For further statistical analysis percent of over- and underestimation was considered. Females tended to assign higher weight to the foods compared to males; however, none of the differences for singe products reached statistical significance after correction for multiple testing (Table 1 and Figure 1A). Similarly, between the different BMI categories, there was a tendency toward a difference (*p* < 0.05) in the recognition of the portion sizes of three types of foods; however, considering the correction for multiple testing and the number of comparisons, there were no statistically significant differences (Table 1 and Figure 1B). The BMI < 18.5 tended to contrast the other BMI groups and there were no consistent differences in the estimation error between the groups. Age and hunger levels each influenced only one estimation (Supplementary Table 2 and Figure 1C). A possible influence between the factors was investigated in a univariate, multifactorial, linear model. There was a combined effect of BMI and gender for two products (fish *F* = 2.650; *p* = 0.048 and butter *F* = 3.288; *p* = 0.021), BMI and age for three foods (noodle *F* = 2.155; *p* = 0.016, berries *F* = 2.350, *p* = 0.008; chips *F* = 1.964, *p* = 0.031), BMI and huger (noodles *F* = 1.947, *p* = 0.045), age and gender (ham *F* = 3.381, *p* = 0.010) each for one product as well as combined effect of gender, BMI, and hunger (rice *F* = 2.120, *p* = 0.034; berry *F* = 2.042, *p* = 0.041; chips *F* = 2.299, *p* = 0.021).

In view of possible interactions between BMI, hunger rating, gender and age, we analyzed the effect of BMI on portion size estimation in a univariate analysis of covariance with the covariates hunger rating, gender and age. For most foods, there were still no significant effects of BMI, with two exceptions: In the corrected model BMI (as continuous variable) had a significant effect on the estimation of the portion size of ham (*F* = 2.954, *P* = 0.001, η^2^ = 0.951) and butter (*F* = 1.797, *P* = 0.008, η^2^ = 0.922). With smaller effect sizes, but still significant, age had an effect on the estimated portion sizes of paprika (*F* = 4.607, *P* = 0.037, η^2^ = 0.008) and pork (*F* = 5.071, *P* = 0.029, η^2^ = 0.096).

In summary, gender, BMI, age, and hunger were factors with a modest impact on the subjects’ capacity to estimate proper portion weight.

### Study II

Study I showed that the participants are not able to estimate the size of a given portion with high accuracy for most foods. However, it was not evident how they view the sizes of the presented foods based on their behavior. Therefore, another group of participants was asked whether they consider different meal sizes as a normal, small, or large portion for their own consumption of these meals. A total of 1,381 individuals participated in the study, 808 of whom completed the questionnaire (Supplementary Figure 1). Further 64 participants were excluded due to misinterpreting the scale and 15 because of an insufficient amount of declared information. A total of 725 individuals completed the questionnaire entirely and correctly and were therefore included in the statistical analysis.

Among the 725 subjects, there were 380 women (51.41%) and 354 (47.59%) men (Supplementary Table 3). The mean BMI of the study population was 25.58 ± 6 kg/m^2^. Underweight participants constitute a very small portion of the samples (2.76%; *N* = 20). The normal weight group accounts for 385 (53.1%) subjects, 201 (27.72%) participants are with overweight and 119 (16.41%) participants are with obesity. The age of the subjects, ranged from 16 to 76 years, with a mean age of 34.14 ± 12 years and a difference between men and women (31.79 vs. 36.92 years; *p* < 0.01). Agreeing with study I, participant’s BMI increased with age (*F* = 1.659, *p* = 0.002) resulting in a significant positive correlation (*r* = 0.16; *p* < 0.001).

Similar to study I, most of the subjects declared feeling full (*N* = 401, 57.78%), and less being somewhat hungry (*N* = 162, 20.78%), moderately hungry (*N* = 112, 15.45%) or very hungry (*N* = 50, 5.98%) (Supplementary Table 3). Men differed significantly in their feeling of hunger from women (*p* = 0.009). The average hunger rating among men was 31.38 ± 26 (on a scale of 1–100, 1–full, 100–very hungry), while that among women was 22.3 ± 26. The hunger rating also differed significantly between the BMI categories (*F* = 1.315, *p* < 0.034) with hunger being reversely correlated to BMI (*r* = –0.93, *p* = 0.013). Thus, at the time of participation, male or low-BMI individuals tended to be more hungry than female or participants with obesity.

According to the Austrian guidelines for a healthy diet, the upper limit of the recommendations of one meat portion is 150 g (Austrian Federal Ministry for Health, 2010). Interestingly, the average ideal portions for goulash exceeded the suggested amount nearly double (297 g) (Supplementary Table 4). However, other meals containing meat (chicken, schnitzel) were within the recommended sizes. Women and men differ in the assessment of their ideal portion size in all but one dish (fruit salad) with women consistently favoring up to 14.66% smaller portions as their ideal portion size (Table 2 and Figure 1D). There was no significant difference within the BMI groups for the total estimation of the portion size. However, there is a clear trend with a tendency to favor bigger portions with increasing BMI (Table 2 and Figure 1E) and the percentage difference in ideal portion sizes of the eight out of 11 dishes were significantly different within the BMI groups (Supplementary Table 4). Importantly, age impacted the portion size estimation of one meal but *p* < 0.05 indicated strong tendencies in discrepancies for six more meals (Supplementary Table 5 and Figure 1F). There was no statistically significant interaction between the factors age and BMI, age, and gender. However, BMI and gender showed a significant interaction for pasta (*F* = 2.868, *p* = 0.036), rice (*F* = 3.390, *p* = 0.018), and kaiserschmarrn (*F* = 2.759, *p* = 0.041). Moreover, hunger perception significantly influenced portion size estimation in five out of 11 dishes (Supplementary Table 6) resulting in an increase in portion size considered as normal with more intensive hunger.

**TABLE 2:** Estimations of perfect portion size in different gender and BMI categories.

	Gender						BMI									
	Female		Male				< 18.5		18.5–25		25–30		> 30			
Meal	Gram ± *SD*	%	Gram ± *SD*	%	*T*	*p*	Gram ± *SD*	%	Gram ± *SD*	%	Gram ± *SD*	%	Gram ± *SD*	%	*F*	*p*
Spaghetti	226.70 ± 86	–10.80	283.95 ± 100	11.84	8.269	**<0.001**	261.00 ± 68	2.78	250.05 ± 95	–1.53	255.97 ± 100	0.80	260.85 ± 102	2.72	0.542	0.653
Chicken	144.43 ± 83	–12.86	206.74 ± 75	14.09	5.177	**<0.001**	146.00 ± 67	–12.00	161.76 ± 74	–2.67	168.66 ± 73	1.48	179.62 ± 73	8.08	0.703	0.550
Salmon	138.23 ± 58	–12.62	180.26 ± 86	13.72	7.738	**<0.001**	140.00 ± 60	–11.57	157.98 ± 78	–0.21	154.04 ± 67	–2.70	169.23 ± 83	6.90	1.378	0.248
Schnitzel	109.39 ± 24	–14.66	148.84 ± 55	15.98	15.02	**<0.001**	107.50 ± 18	–16.16	128.39 ± 48	–0.13	128.86 ± 45	0.50	130.25 ± 42	1.59	1.445	0.229
Pizza	296.44 ± 92	–10.03	375.28 ± 187	11.01	7.298	**<0.001**	307.35 ± 87	–6.59	323.50 ± 94	–1.68	332.17 ± 87	0.95	346.14 ± 82	5.20	1.862	0.135
Goulash	275.19 ± 100	–7.46	321.66 ± 109	8.17	5.970	**<0.001**	265.00 ± 67	–10.87	293.12 ± 108	–1.41	303.75 ± 108	2.17	304.62 ± 106	2.46	1.274	0.282
Rice	349.87 ± 92	–5.04	388.37 ± 101	5.54	5.356	**<0.001**	355.00 ± 89	–3.58	368.01 ± 97	–0.04	362.50 ± 98	–1.54	380.25 ± 104	3.28	0.983	0.422
French fries	144.81 ± 56	–9.74	183.58 ± 145	10.58	4.776	**<0.001**	157.50 ± 51	–1.59	159.59 ± 64	–0.29	161.63 ± 61	–0.98	159.24 ± 55	–0.51	0.132	0.941
Kaiserschmarrn	199.18 ± 52	–3.05	212.22 ± 56	3.36	3.260	**0.001**	220.00 ± 47	7.13	206.36 ± 56	0.49	199.75 ± 52	–2.74	209.32 ± 52	1.93	1.345	0.259
Fruit salad	190.92 ± 124	–3.78	216.43 ± 130	4.16	1.737	0.083	204.75 ± 40	1.23	203.51 ± 75	0.61	207.81 ± 72	2.74	188.81 ± 69	–6.65	1.224	0.300
Ice cream	68.91 ± 30	–10.35	85.64 ± 36	11.28	6.746	**<0.001**	73.50 ± 27	–4.41	75.27 ± 335	–2.11	78.66 ± 34	2.22	79.66 ± 34	3.50	0.760	0.517
Average		–9.13		9.98				–5.06		–0.79		–0.44		2.60		

*The data represents ideal portion estimations in gram and in% change compared to the estimations of the whole population. Bold values indicate statistically significant differences.*

Similar to study I, the effect of BMI on ideal portion size was analyzed in a univariate analysis of covariance with the covariates hunger rating, gender and age. After controlling for these covariates, again no significant effects of BMI on ideal portion sizes could be found.

The average scores of the DEBQ of all participants are highest in the category of externally determined dietary behavior (3.13). The mean score of restrictive eating behavior is 2.46 and that of emotional eating behavior is 2.09. Therefore, the participants of this study agree most with the statements of externally determined eating behavior. When analyzing ideal portion sizes including hunger rating, gender, age and BMI as covariates, estimates of four dishes were affected by emotional and seven by restrictive as well as externally motivated eating behavior traits indicating a strong impact of eating behavior on meal size choices (Table 3). Sizes of all dishes correlated negatively with restrictive and positively with emotional and externally motivated traits. In general, smaller portions of meals were chosen by participants with a tendency to be affected by restrictive behavior, and bigger portions of all of the meals were preferred by subjects showing a tendency to be affected by external cues. Emotional eating behaviors did not have a consistent impact on portion size estimation. The internal consistency of the subscales was within acceptable span (Cronbach’s alpha within the range 0.7–0.8) for both groups emotional and non-emotional eating behaviors as well as non-externally motivated and non-restrictive individuals. However, the consistency was lower (Cronbach’s alpha within the range 0.6–0.7) in the case of externally motivated and restrictive subjects.

**TABLE 3:** Estimations of perfect portion size in different DEBQ categories by partial correlations with the hunger, gender, age, and BMI as control variables.

	Restrictive		Emotional		External	
Meal	*r*	Sig.	*r*	Sig.	*r*	Sig.
Spaghetti	–0.099	**0.008**	0.067	0.074	0.096	**0.011**
Chicken	–0.029	0.433	0.051	0.176	0.068	0.067
Salmon	–0.137	**<0.000**	0.073	0.052	0.042	0.265
Schnitzel	–0.087	**0.020**	0.031	0.416	0.068	0.070
Pizza	–0.085	**0.023**	0.088	**0.018**	0.153	**0.000**
Goulash	–0.088	**0.019**	0.051	0.174	0.108	**0.004**
Rice	–0.134	**<0.000**	0.098	**0.009**	0.152	**0.000**
French fries	–0.053	0.157	0.053	0.160	0.140	**0.000**
Kaiserschmarrn	–0.029	0.438	0.165	**0.000**	0.194	**0.000**
Fruit salad	–0.020	0.593	0.021	0.583	0.037	0.318
Ice cream	–0.072	0.055	0.090	**0.016**	0.087	**0.020**

*The data represents the correlation and the associated p-values. Bold values indicate statistically significant differences.*

## Discussion

The correct estimation of the portion size of foods is important for nutritional surveys, as foods are frequently reported based on standard portion sizes and not by their exact or estimated weight. The participants’ ability to respond to questions on the amount of food consumed depends on their estimate of the weight of the corresponding food. When a participant in a nutrition survey considers the portion size of food larger or smaller than the standard portion size, this will lead to over- or underestimation of the contribution of this food to the intake of associated nutrients, including energy. Consequently, it is important to understand which factors have an impact on the ability of portion size and food weight estimation. We hypothesized, that a high BMI results in an underestimation of a portion for a given food, which in turn will result in an underreporting of the overall amount of food consumed. We did observe deviations of the estimates from the actual food weights, depending on the food shown, but these deviations were not strongly influenced by age, BMI, and even less by age or hunger.

Overall, we concluded from the study I that participants are not well trained in estimating the weight of foods presented in form of pictures. Particularly problematic appeared estimation of high-calorie foods chips and butter, which should be concerning. It is possible, that participants are better able to estimate food weights in real life and that the artificial setting of the foods had an impact on the estimates. Since the setting was comparable for all food pictures, we believe that this had only a minor influence on our results. BMI had a small impact on the portion weight estimates, while the type of food had a strong effect.

As we found some impact of the BMI on the food weight estimates, we tried to assess whether this is also the case for the subjective rating of portion size, i.e., for the portion size considered as a normal size from the individual’s point of view. Here, we hypothesized that subjects with a higher BMI would consider a larger portion of a given food as their normal size compared to subjects at a lower BMI. To study this, we prepared in our laboratory popular dishes and meals which varied the size of one part of the meal or the size of the dish and asked whether this portion in the given setting is larger or smaller than the normally consumed portion of the interviewees. We deliberately selected some meals with various components (e.g., a schnitzel with potato salad and a side salad) to reduce artificiality from the setting. For these pictures, we varied only one component and asked the participants to select their normal portion size of this component at the given setting.

We showed that BMI, contrary to gender, had very little impact on ideal portion size estimation. For all but one of the foods tested, female participants selected significantly lower portion sizes than male participants. Our results concerning both BMI and gender are in line with other reports using a similar study design (Brunstrom et al., 2008). The physiologically higher energy requirement of men compared to women (Klausen et al., 1997) is reflected in the assessment of larger ideal portions in all dishes examined. Additionally, gender stereotypes in nutrition can impact portion-related decisions. For example, the size of the portions also serves as a sign of gender identity. Women, especially in male company, deliberately choose smaller portions of lower-calorie food, while men prefer large portions of “masculine” food (Rozin et al., 2003; Young et al., 2009; Cavazza et al., 2017). Furthermore, foods such as vegetables, white meat, fish, or dairy products are considered feminine, while red meat is considered particularly masculine (O’Doherty Jensen and Holm, 1999).

Despite the estimation raising with increasing BMI, BMI did not affect normal portion sizes statistically significantly. Previous studies show contradictory results (Dodd et al., 1976; Burger et al., 2007; Brunstrom et al., 2008; Lewis et al., 2015; Reily et al., 2016; Embling et al., 2021). Likely, the presentation style and type depicted foods play a role in the outcomes as the study from Burger et al. (2007) reported a positive relationship between BMI and some of the foods tested (peanuts, M&M candies, cereals, jam, and soda), but also some foods with a negative relationship (margarine and apple sauce) and some with no differences (rice, chips, peanut butter, macaroni and cheese, and water). In our study, the differences between BMI groups, however, were observed only for the percentage difference from average and were mainly driven by the lowest BMI group (<18.5 kg/m^2^) with smaller portion sizes than the higher BMI categories. Overall, the results from our study show that gender has a stronger impact than BMI concerning portion sizes.

Similar to gender, age had a uniform effect on the portion sizes considered as normal, typically with lower portion sizes with increasing age. The age-associated changes in caloric needs are reflected in the participants’ choices of perfect portion size. Literature confirms this finding and suggests that younger adults assess portion size more accurately than older adults with older adults tending to overestimate food portion size (Young and Nestle, 1995; Nelson et al., 1996). However, in our study, the trend in stronger over- or underestimation between different age groups was not linear.

In experiments associated with portion sizes that are executed under laboratory conditions, participants are instructed to consume the exact amount of food at a specified time before the investigation. Since this standardization is not possible in online surveys, the participants were asked to declare a subjective feeling of hunger. Not surprisingly, hungry people tended to assess their ideal portions higher than people who claim to be full. The observation that hunger is associated with larger preferred portions is justified in the literature with the so-called energy compensation. The size of the last meal and the time that elapses before the next meal, therefore, influences the portion size of the following meal (Benton, 2015). In both studies, women stated more often to be full or somewhat hungry, whereas men were more often hungry. Importantly, the assessment of portion sizes in an online questionnaire does not necessarily coincide with the amount actually consumed. For this purpose, real experiments in which participants consume their chosen ideal portion must be performed (Polivy and Herman, 2017).

Restrictive dietary behavior is associated with a tendency to lower energy intake and with smaller, low-energy meals (Brunstrom et al., 2008; Olea Lopez and Johnson, 2016). Accordingly, our study depicts the important role of restrictive behaviors and in line with previous studies indicates the choice of smaller proffered portions by individuals with restrictive tendencies. Furthermore, externally determined dietary behavior impacts the estimation of the ideal portion. Contrary, emotionally induced behaviors play a minor role. Previously, eating style has been shown to influence choices of favorable (healthy) and unfavorable (unhealthy) foods (Malachowska et al., 2021), however, in our study all types of foods seemed to be affected equally.

The here presented approach using freshly cooked and multiple-ingredient complex dishes distinguishes study II from previous surveys where often individual foods or easily portioned, ready-to-serve meals such as toast or snacks are usually used to investigate the subjective assessment of portion sizes (Almiron-Roig et al., 2013; Lewis et al., 2015; Faulkner et al., 2016). Despite this, study II is accompanied by some limitations. The collection of the subjective assessment of portion sizes is subject to several sources of error. Although the survey was anonymous, influencing factors such as social norm sizes and underreporting can be of great importance, especially for persons with obesity or overweight (Almiron-Roig et al., 2013; Lewis et al., 2015). Although these forms of bias can be counteracted by filling out the questionnaire anonymously, they cannot be prevented. Furthermore, the connection between social norm sizes and personal ideal portions could be investigated with another question, as to how the general public would assess the ideal portion size of the respective dish (Lewis et al., 2015). Given the already complex questionnaire structure, however, this aspect was omitted.

Considering the approach adopted by us, screen-based estimation carries a risk of inaccuracy due to the artificiality of the conditions. However, previous studies prove the validity of such approach indicating expected satiety ideal portion size and liking in screen-based set up as a good predictor of actual food intake (Wilkinson et al., 2012). Online and in-person approaches all have their limitations. In the case of online surveys, technical demands including usage of certain screen quality and size which is difficult to control for. In our study, in order to increase participation, we did not restrict screen parameters but we specified picture size for all types of screens. Also, various types of online surveys are used to estimate portion sizes (Embling et al., 2021; Pink and Cheon, 2021). However, when compared, for example, simplified and standard portion selection task forms both results in comparable results and can be used online to estimate ideal portion size (Pink and Cheon, 2021).

Our design of study II is directly comparable to the experiments by Brunstrom et al. (2008) with the idea of presenting portions and asking to indicate whether it is larger or smaller than the participants’ usual portion as well as data being stratified by gender, BMI, and reported hunger level. However, in our approach, participants with wide range of ages were included and the number of pictures of each food was strongly reduced to increase the contrast between the presented portions and obtain clearer participants’ answers. Despite the differences in the design, the overall results of the studies were convergent in terms of the impact of gender, BMI, hunger, and dietary behaviors. However, we generally measured a stronger impact (statistically significant differences for a higher number of foods) which is likely connected with the difference in choice of presented foods.

Finally, it is important to note, that the portion size regarded as normal for a given individual is affected not only by internal factors as tested in our study. In particular, the “normalizing” effect of reduced portion sizes has been reported already by Robinson and Kersbergen (2018) and Robinson et al. (2019), while other studies showed increasing portion sizes in different settings over time leading to portion distortion effects (Young and Nestle, 2003; Young, 2005; Schwartz and Byrd-Bredbenner, 2006). In view of the shift in portion sizes toward larger portions over the past years, a better understanding of long-term effects on shaping perceived portion sizes needs further study (Robinson et al., 2016).

Gender has a significant influence on weight recognition and the assessment of ideal portion sizes with men consistently judging their ideal portions to be larger than women. Contrary to our hypothesis, BMI has a low impact on ideal portion size. However, bias sources such as socially desirable portion sizes and underreporting must be accounted for. Importantly, the portion choice does not directly reflect energy intake. Our analysis indicates that the factors gender, externally determined, and restrictive dietary behavior, age, and hunger, are the main predictors of portion size selection, and BMI a relatively weak predictor.

## Data Availability Statement

The raw data supporting the conclusions of this article will be made available by the authors, without undue reservation.

## Ethics Statement

The studies involving human participants were reviewed and approved by the Ethics Committee of the University of Vienna. The patients/participants provided their written informed consent to participate in this study.

## Author Contributions

KD analyzed the data and wrote the manuscript. MH designed and performed study II. ID and DK designed and performed study I. JK designed and supervised the studies. All authors contributed to the article and approved the submitted version.

## Conflict of Interest

The authors declare that the research was conducted in the absence of any commercial or financial relationships that could be construed as a potential conflict of interest.

## Publisher’s Note

All claims expressed in this article are solely those of the authors and do not necessarily represent those of their affiliated organizations, or those of the publisher, the editors and the reviewers. Any product that may be evaluated in this article, or claim that may be made by its manufacturer, is not guaranteed or endorsed by the publisher.

## Footnotes

1
https://www.soscisurvey.de
