# Supplementary figures and images for: Gender, Age, Hunger, and Body Mass Index as Factors Influencing Portion Size Estimation and Ideal Portion Sizes
Source: Front Psychol. 2022 May 11;13:873835. doi: 10.3389/fpsyg.2022.873835 (PMC9130823; doi:10.3389/fpsyg.2022.873835)

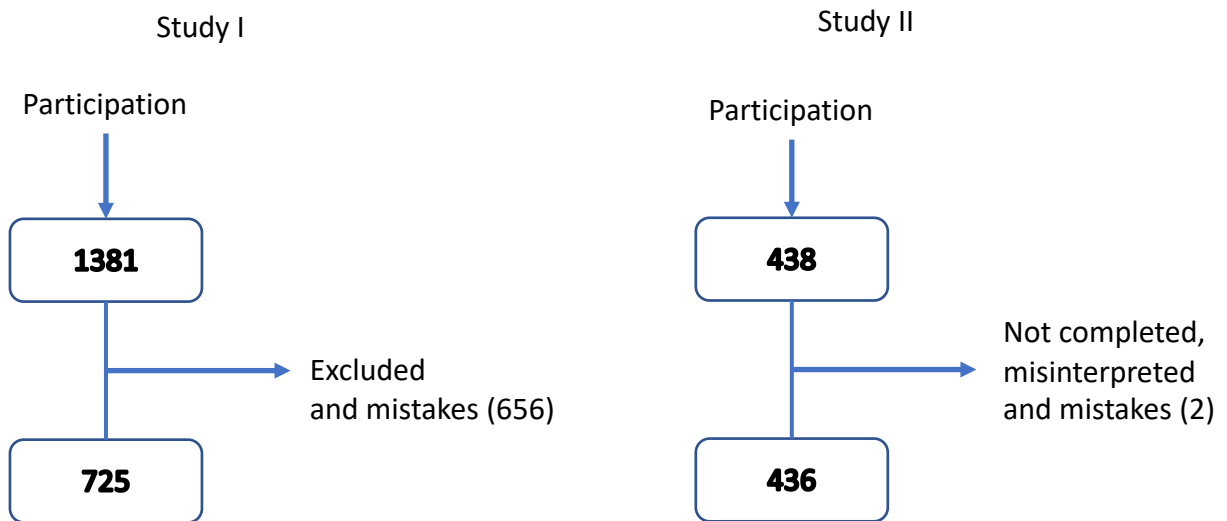

Supplementary Figure 1. Outline of study I and study II

Supplement: Supplementary file 1 [file Data_Sheet_1.PDF]

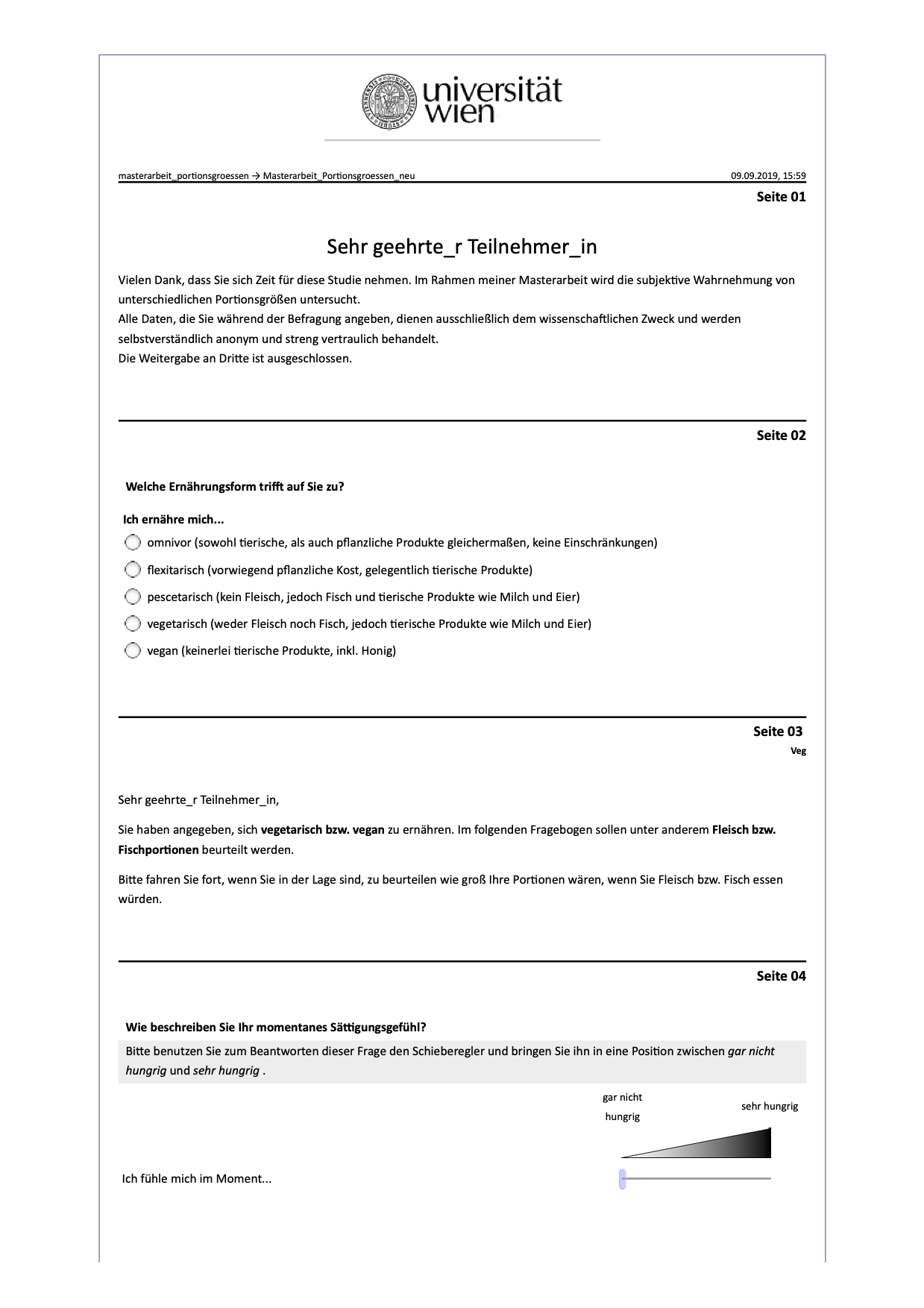

Supplement: Supplementary file 4 [file Image_1.PNG]
