# Supplementary material for: Gender, Age, Hunger, and Body Mass Index as Factors Influencing Portion Size Estimation and Ideal Portion Sizes
Source: Front Psychol. 2022 May 11;13:873835. doi: 10.3389/fpsyg.2022.873835 (PMC9130823; doi:10.3389/fpsyg.2022.873835)
Supplement: Supplementary file 2 [file Data_Sheet_2.PDF]

Umfrage Abschätzung von Portionsgrößen

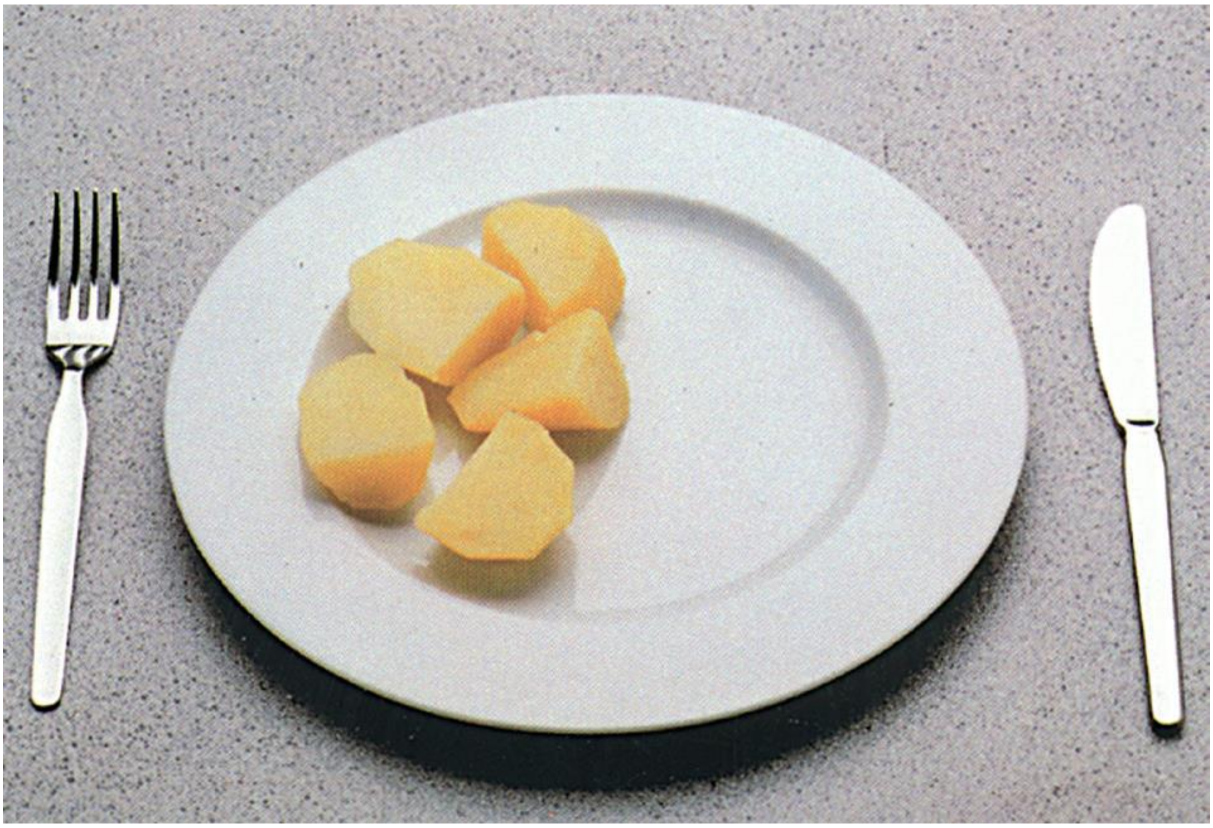

001-2

Geben Sie eine Schätzung für diese Portion Salzkartoffeln in Gramm an:

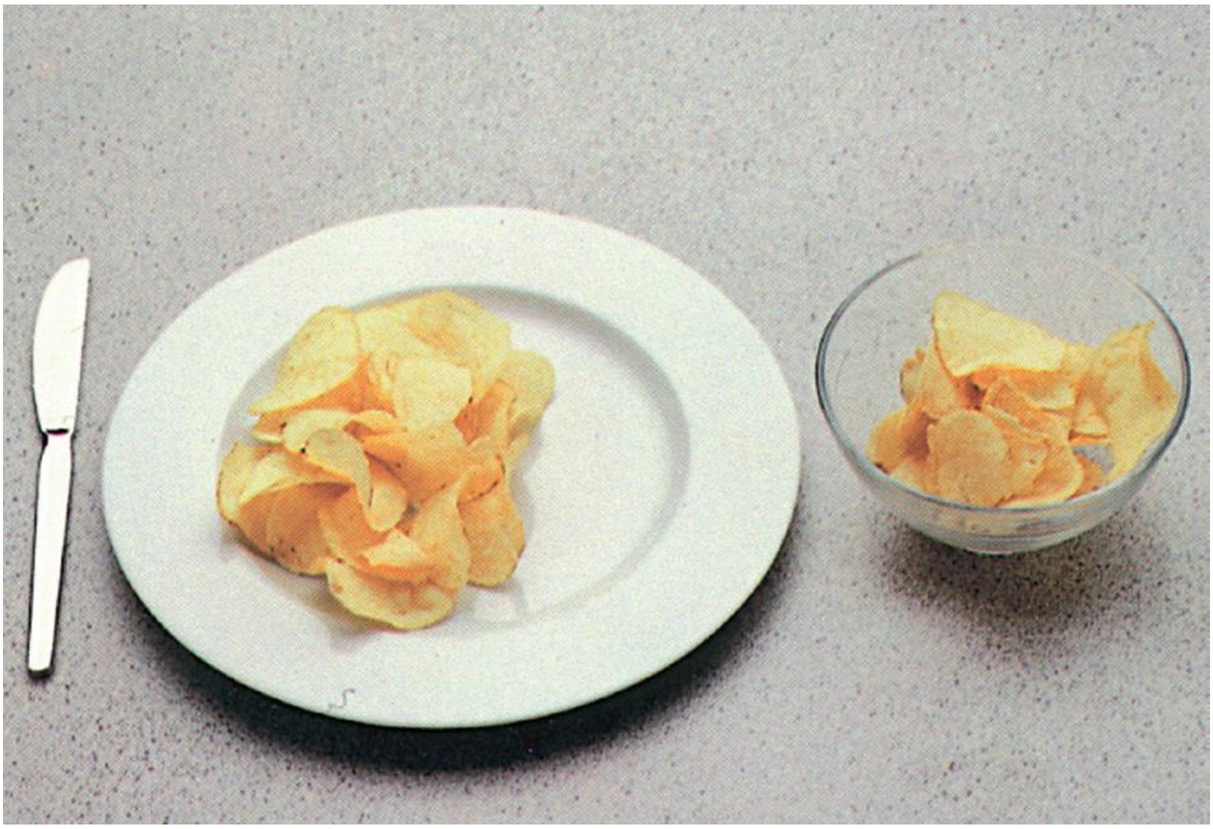

005-2

Geben Sie eine Schätzung für diese Portion Kartoffelchips (am weißen Teller) in Gramm an:

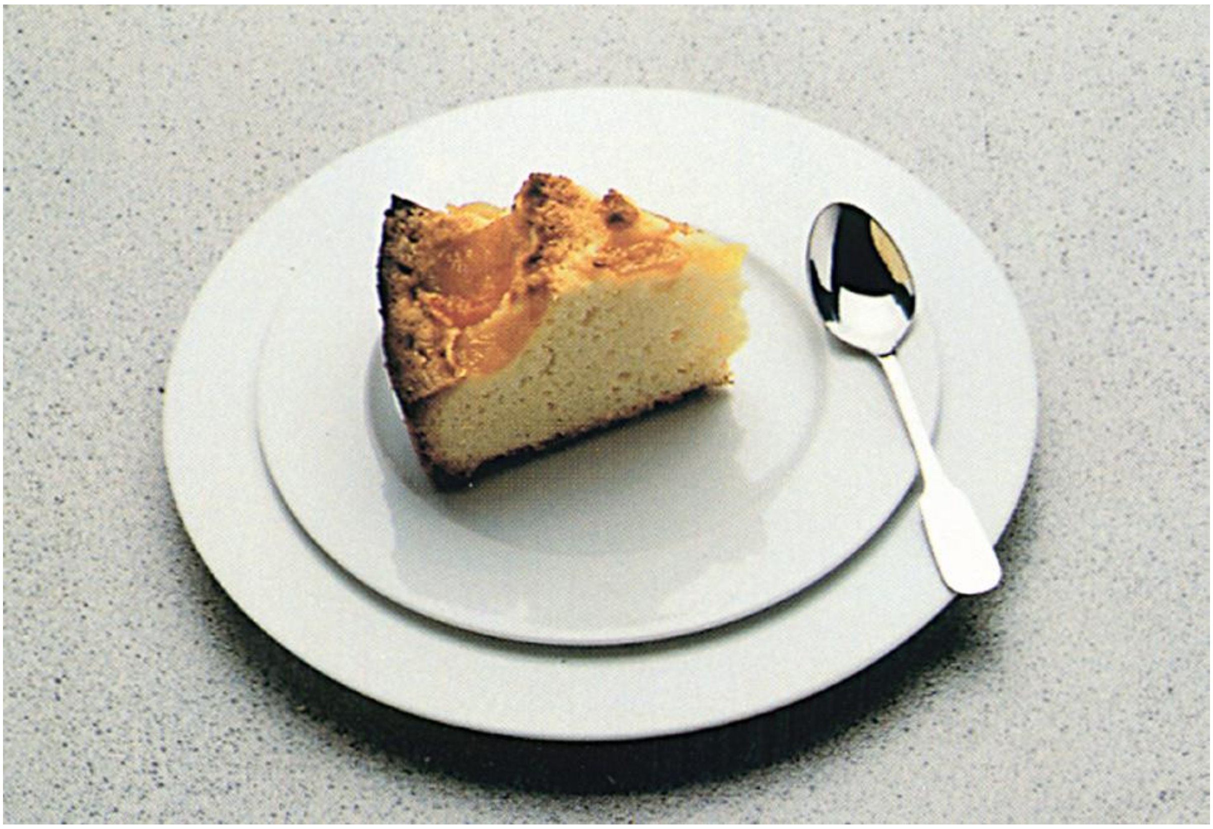

064-3

Geben Sie eine Schätzung für diese Portion Früchtekuchen in Gramm an:

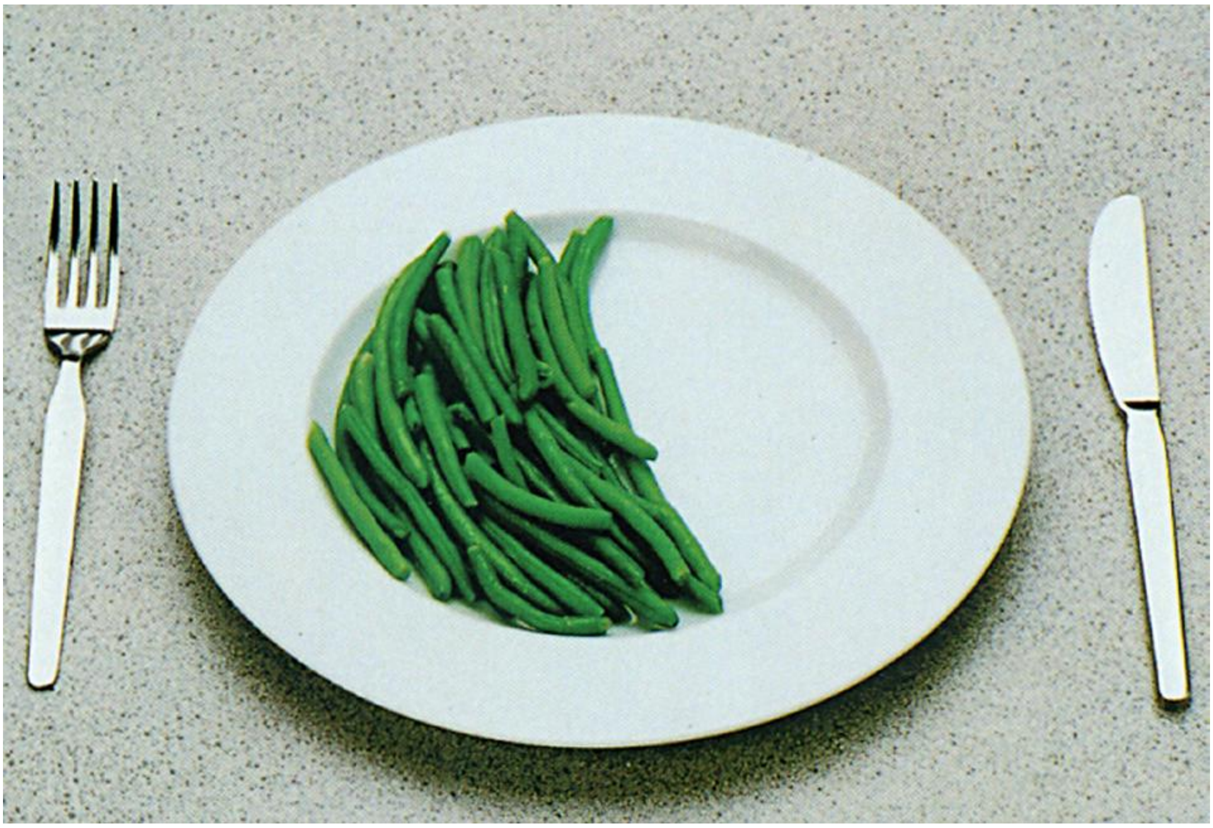

010-2

Geben Sie eine Schätzung für diese Portion Fisolen in Gramm an:

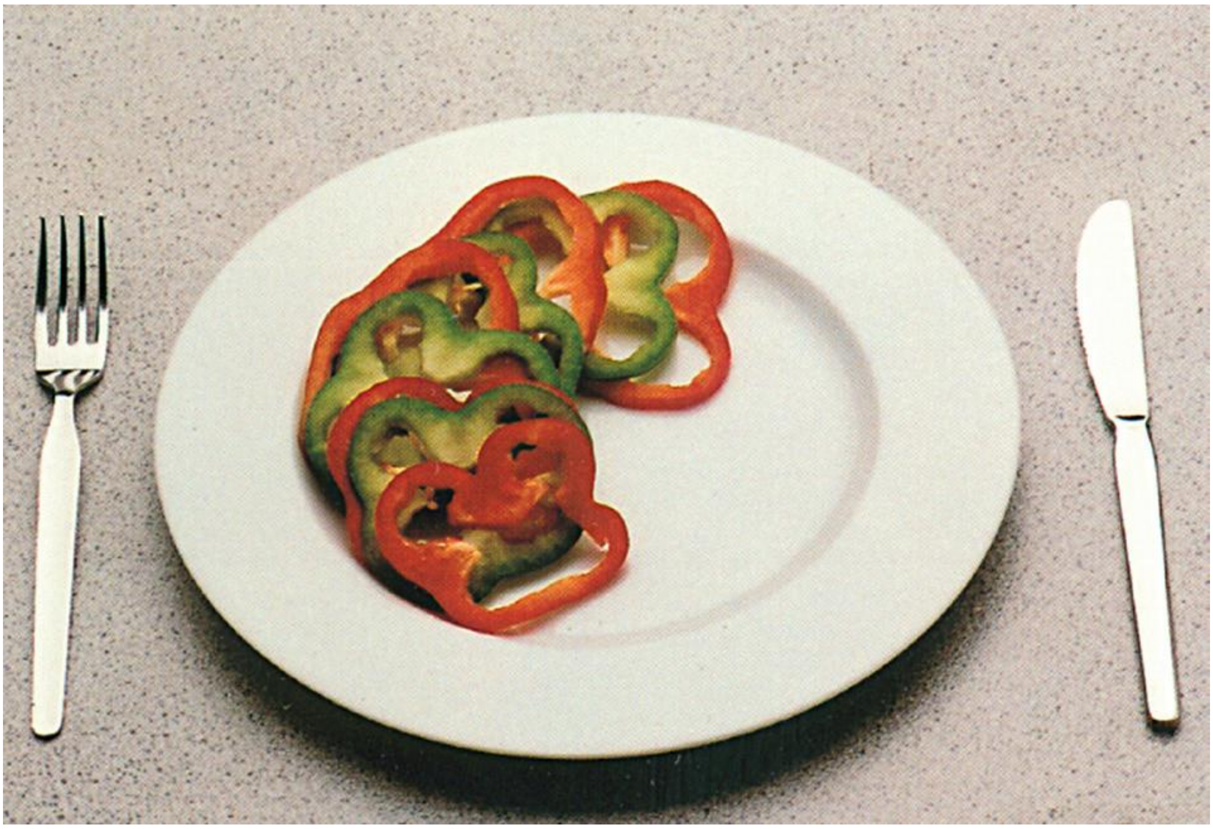

014-3
Geben Sie eine Schätzung für diese Portion Paprika in Gramm an:

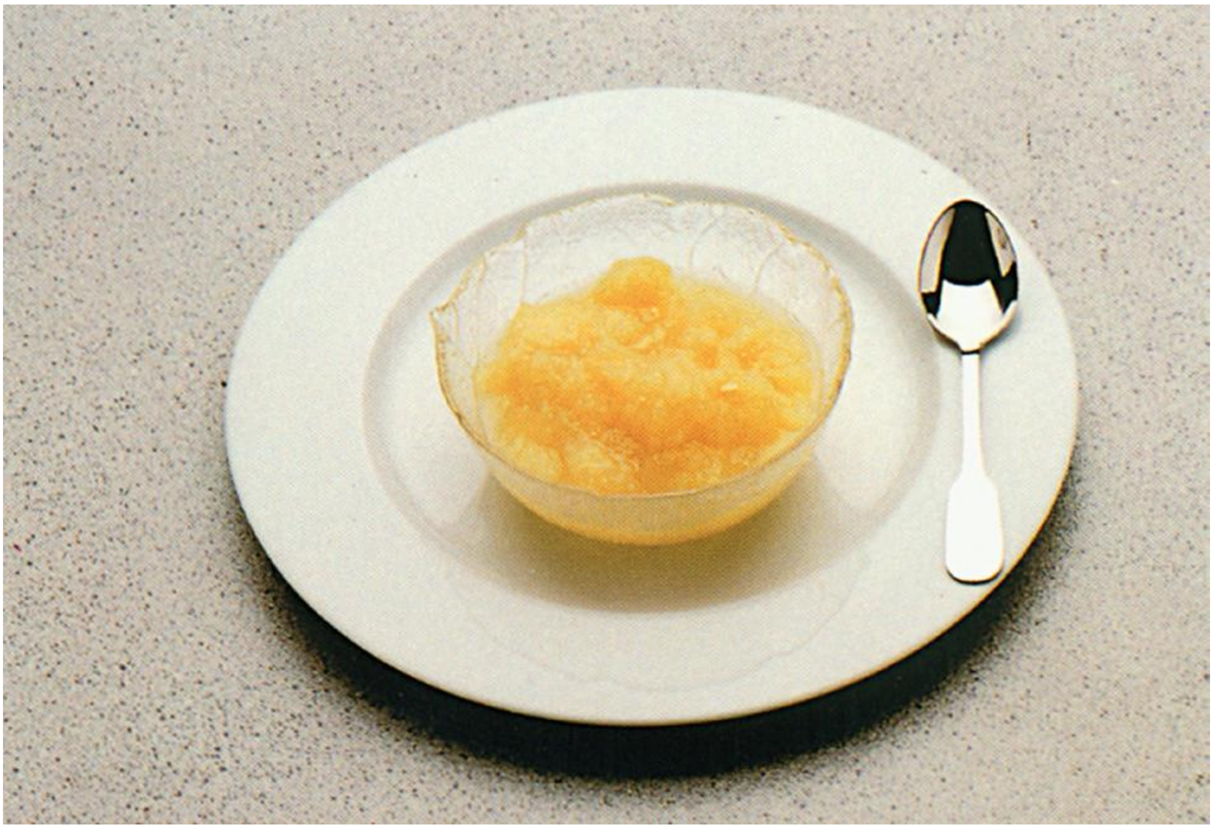

025-4
Geben Sie eine Schätzung für diese Portion Apfelmus in Gramm an:

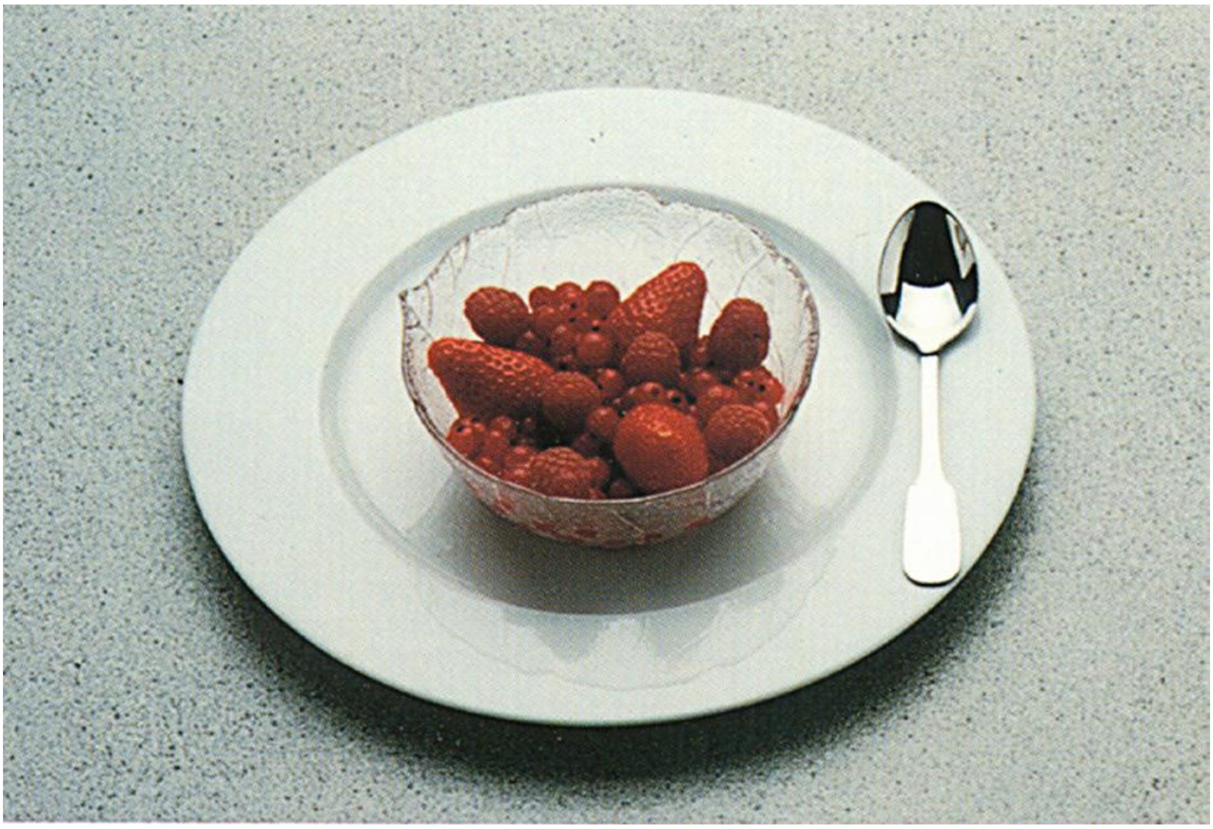

026-3
Geben Sie eine Schätzung für diese Portion Beerenmix in Gramm an:

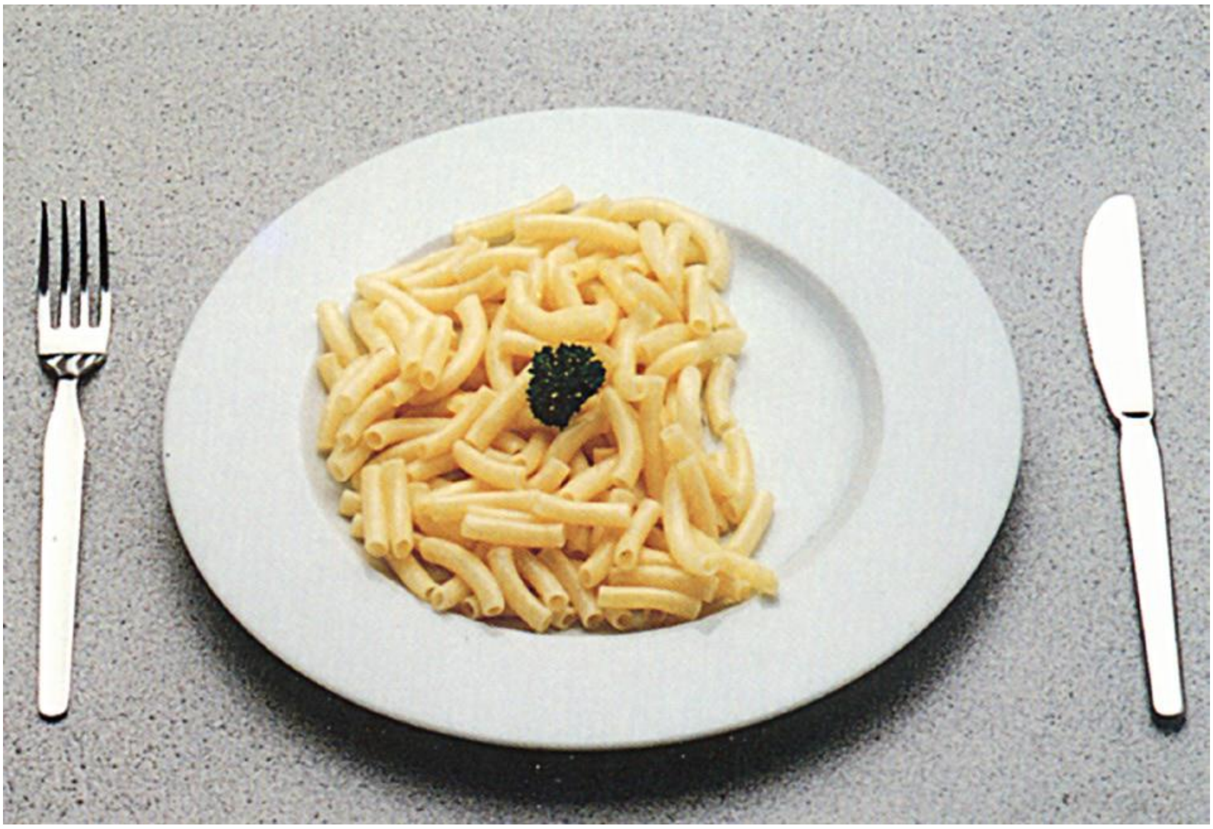

034-2
Geben Sie eine Schätzung für diese Portion Nudeln in Gramm an:

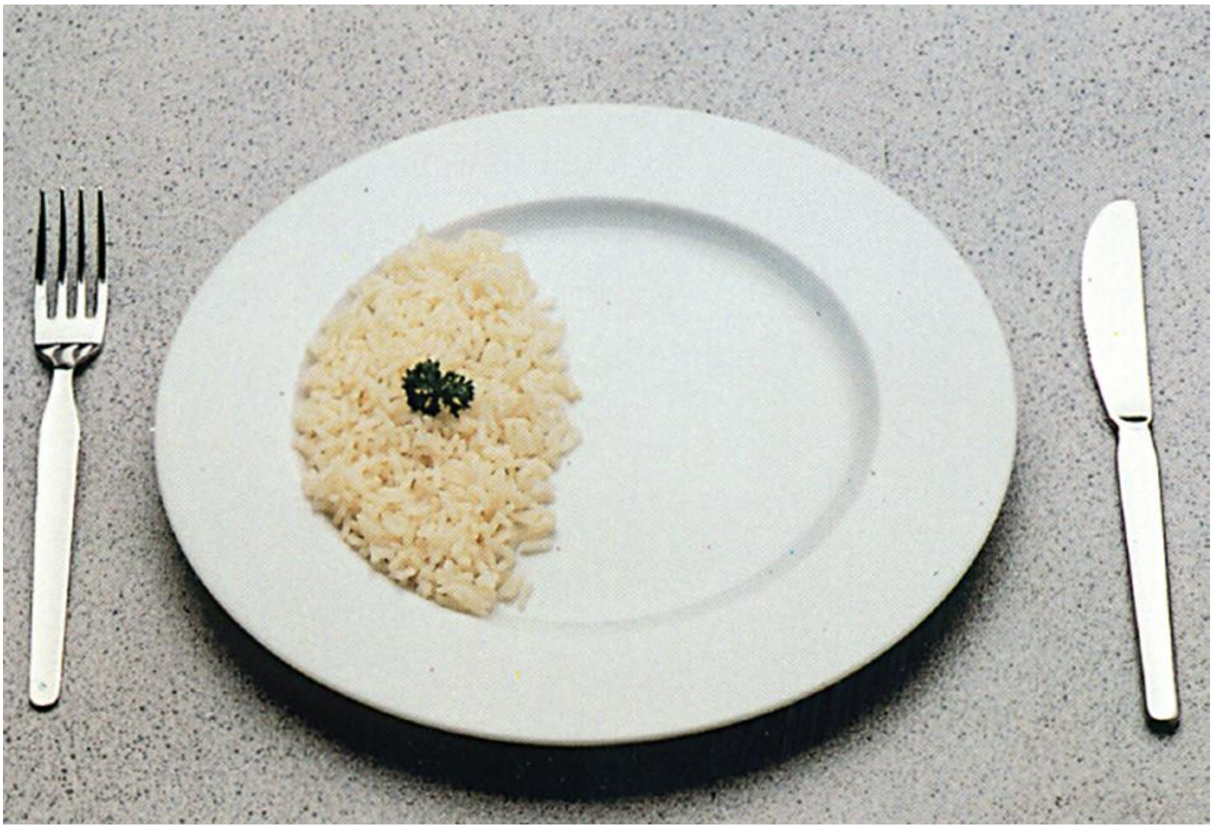

036-1
Geben Sie eine Schätzung für diese Portion Reis in Gramm an:

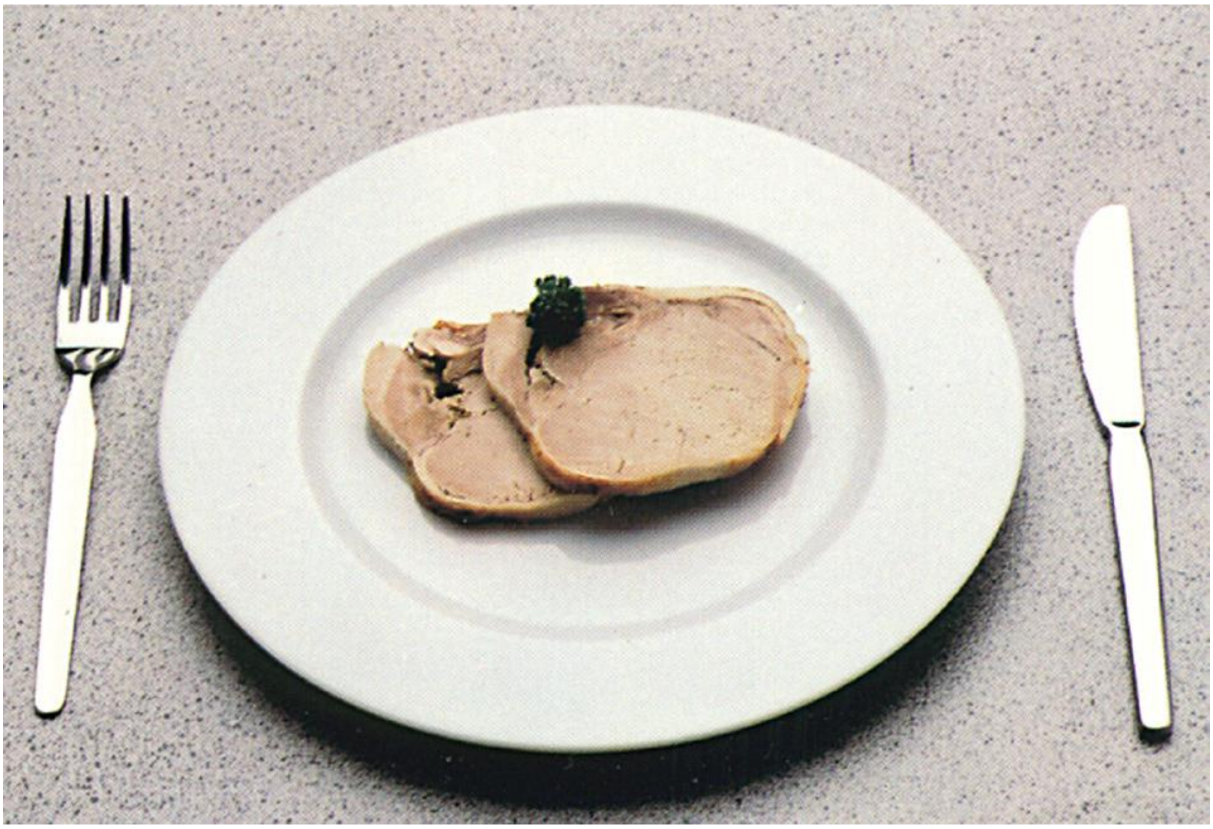

043-1
Geben Sie eine Schätzung für diese Portion Schweinsbraten in Gramm an:

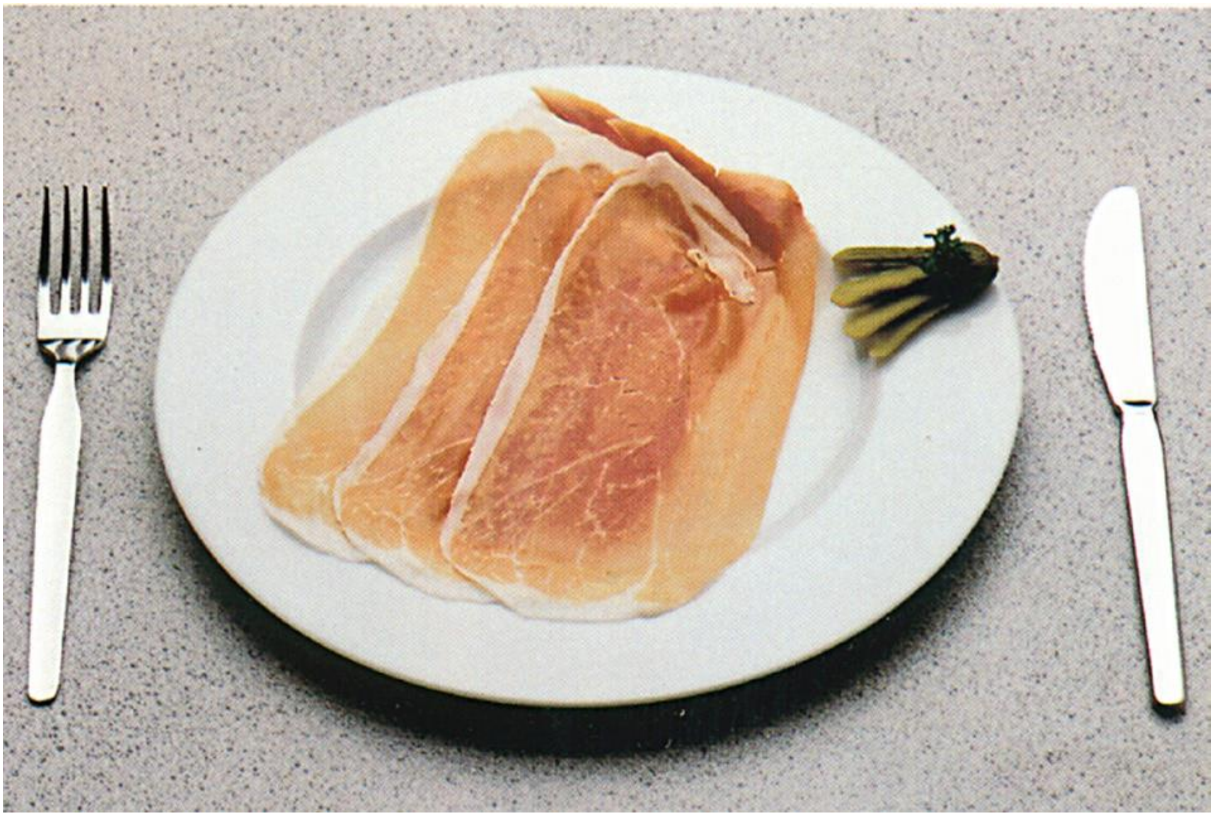

050-3

Geben Sie eine Schätzung für diese Portion Rohschinken in Gramm an:

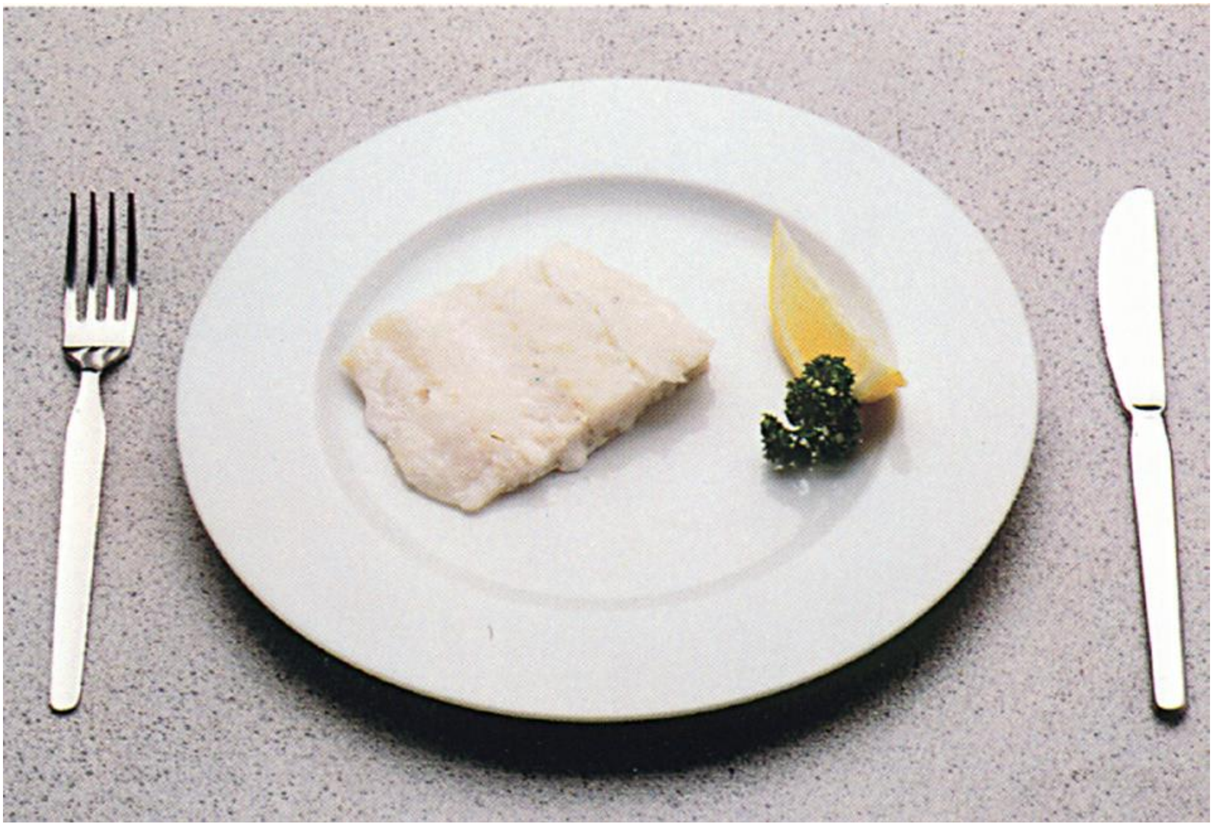

,

051-2

Geben Sie eine Schätzung für diese Portion Fischfilet in Gramm an:

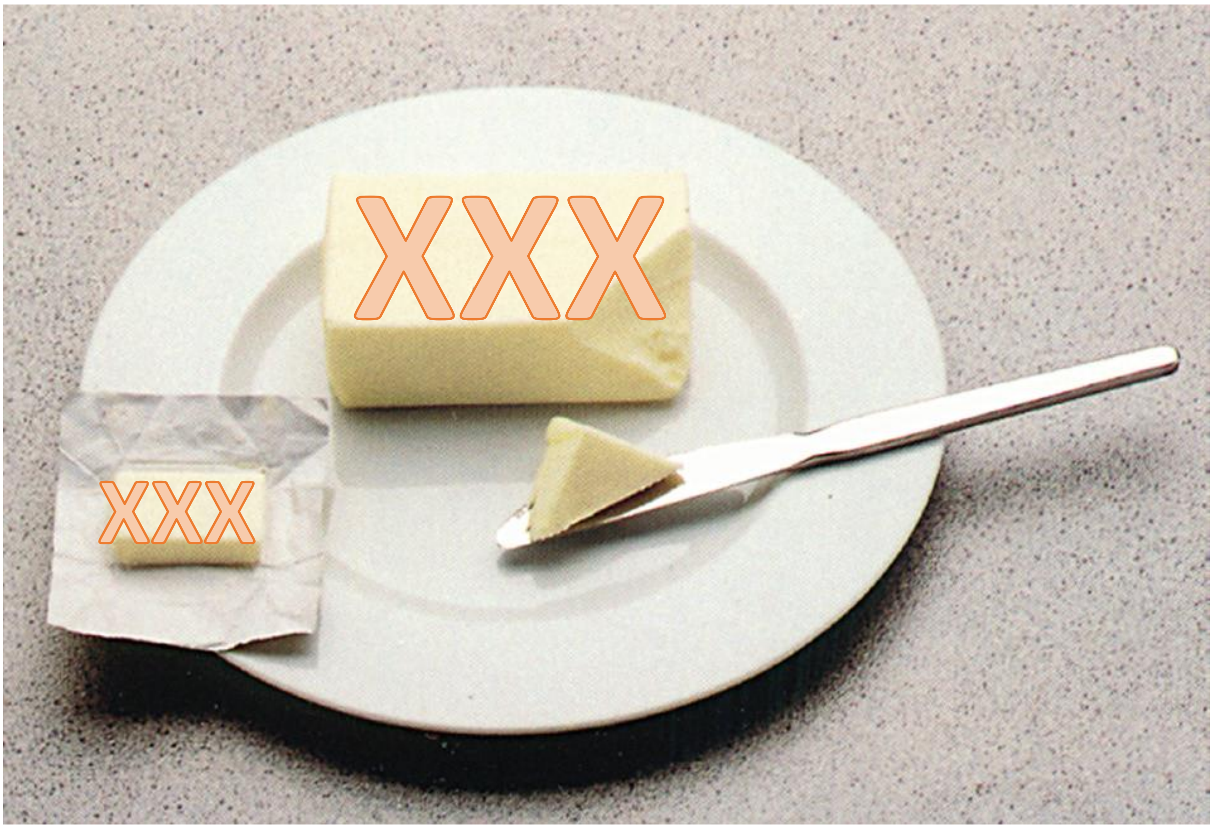

057-3

Geben Sie eine Schätzung für diese Portion Butter (am Messer) in Gramm an:

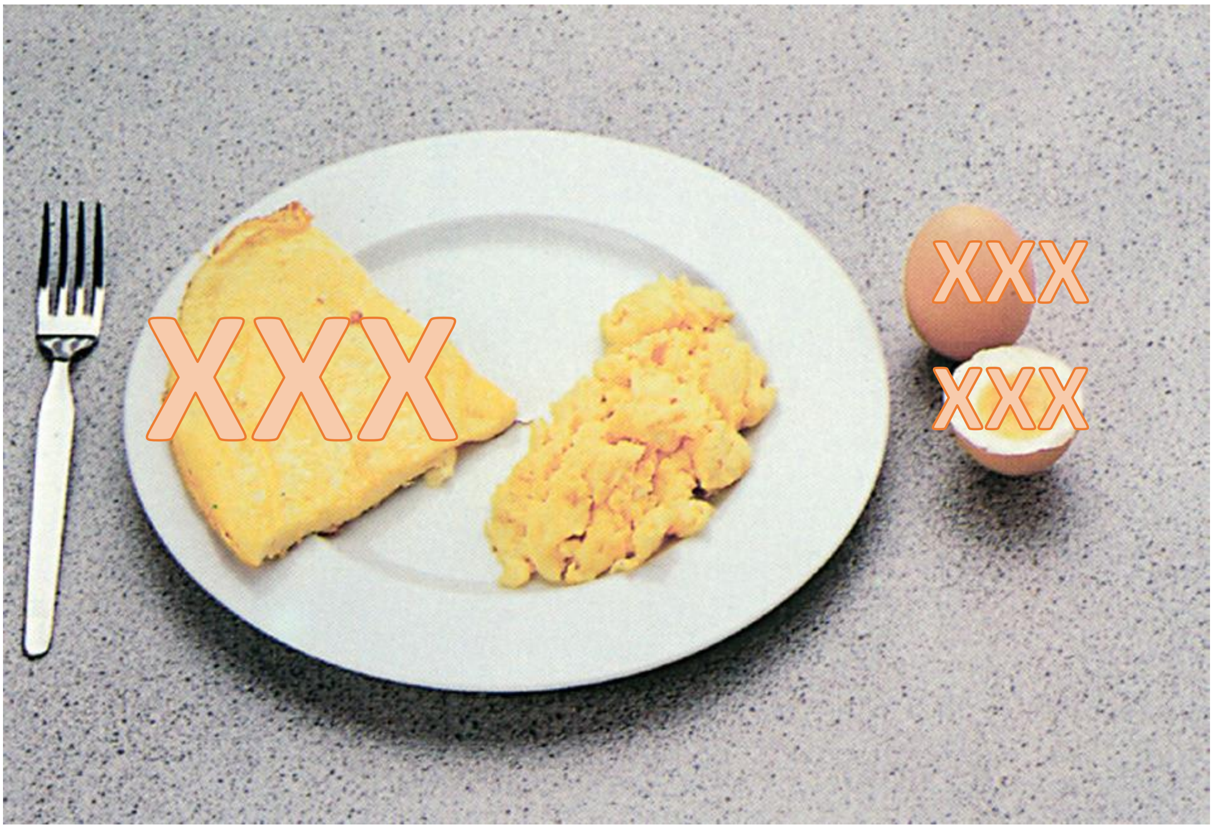

302-2

Geben Sie eine Schätzung für diese Portion Rührei (nicht das Omelette) in Gramm an:

Wie alt sind Sie (in Jahren):

Wie viel wiegen Sie (in Kilogramm, ganze Zahl):

Wie groß sind Sie (in cm, ganze Zahl):

Sind sie männlich oder weiblich: (Dropdownmenü)

Wie würden Sie ihre eigenen Fähigkeiten zur Abschätzung beschreiben: (gut/mittel/schlecht)

Welchen Beruf üben Sie aus?

Gestaltung online:

- Limitation auf 3 Ziffern bei den Abschätzungen in der Umfrage um Kommastellen und Texte zu unterbinden.
- In der Umfrage wird natürlich die Bezugsziffer nicht angegeben, die dient nur der Auswertung und Zuordnung.
- Einleitung als Zielsetzung und Durchführung mit Zeitangabe am Anfang
- Am Schluss noch ein kleines Goodie für die Teilnehmer. Eventuell die richtigen Werte der Portionen per Mail am Ende der Umfrage.
- Ein Beispiel bei der Einleitung mit vorgegebenen Gramm wäre eventuell anzudenken als anfängliche Orientierungshilfe.
