# Supplementary material for: Gender, Age, Hunger, and Body Mass Index as Factors Influencing Portion Size Estimation and Ideal Portion Sizes
Source: Front Psychol. 2022 May 11;13:873835. doi: 10.3389/fpsyg.2022.873835 (PMC9130823; doi:10.3389/fpsyg.2022.873835)
Supplement: Supplementary file 3 [file Data_Sheet_3.PDF]

Supplementary methods

1. Study I

1.1. Assessment of Portion Sizes

The questionnaire consisted of photographs of foods that were provided by the Department of Nutritional Sciences at the University of Vienna (available as Supplementary files) and originated from the Photo Book used for the portion size assessment for the Austrian Nutrition Survey [1]. For about 96 different foods, this book shows 4-6 pictures with increasing portion sizes and known exact weight of these foods shown. We selected 14 different foods across all food groups and picked the medium portion sizes, e.g. the second or third portion size (depending on the number of available pictures for each food). Each picture showed a specified, weighted portion of one of the following foods: boiled potatoes, potato chips, cake, beans, paprika, apple compote, a mix of berries, noodles, rice, pork meat, ham, fish filet, butter, and scrambled eggs. The participants were asked: "Please give the estimation of the portion of (name of the food presented in the picture) in gram".

2. Study II

2.1. Portions preparation

The following meals were prepared, weighed, and documented via photographs (pictures are available in the Supplementary data file) at the Department of Nutrition at the University of Vienna: spaghetti with tomato sauce (referred to as spaghetti), chicken filet with rice and vegetables (referred to as chicken), salmon with vegetables and mashed potatoes (referred to as salmon), Viennese schnitzel with a mixed green salad and potato salad (referred to as Schnitzel), pizza, goulash with a bun (referred to as goulash), vegetable rice pan (referred to as rice), french fries, kaiserschmarrn with plum marmalade (referred to as Kaiserschmarrn), fruit salad, and vanilla ice cream (referred to as ice cream). The meals were chosen based on Austrian cuisine preferences from Statista (https://de.statista.com). Between four and eight photographs were made per meal to depict different portion sizes. To ensure that external distractions were as low as possible, the meals were presented on simple white plates and if possible, plates of the same size. Some of the meals were served on smaller plates for aesthetic reasons. Chicken, salmon, schnitzel, pizza, rice, and French fries were all served on a large plate with a 26cm diameter. Spaghetti, goulash, kaiserschmarrn, and fruit salad were served on a deep plate with a 20cm diameter, whereas the ice cream was served on a flat plate with a diameter of 20cm. To assist in estimating plate sizes, a knife, fork, and spoon were placed next to the plate. For each meal, the portion sizes were presented randomly (i.e. not arranged according to size) during the questionnaire. Every photograph was individually depicted in the questionnaire. For some of the meals (chicken, salmon, and schnitzel) only the main component was modified while the side dishes were constant for all plates. The intention was to ask for the preferred portion size during a typical eating occasion of a meal. The survey was aimed at inhabitants of Austria and for that reason, the pictures presented regionally recognizable typical foods. The foods were chosen based on the familiarity in Austria as assessed by Statista Research Department by a survey on the most popular foods in Austrian in 2016 (Statista GmbH, www.statista.at).

2.2. Assessment of Portion Sizes

Since the category of the dish can influence the estimation of the portion size [2], the description whether it is the main course or a dessert as well as the name of the dishes was labeled on top of the photograph. Underneath the photograph following labels were added: 1) A reference that only the amount of meat or fish varies in dishes with more than one component, whereas the other components remained the same; 2) An instruction on how to use the visual analog scale ("Please rate how the shown portion size differs from your normal portion. Use the slide, if this is in the middle of the scale, the shown portion size represents your normal portion."); 3) A linear scale including the beginning of the following sentence "In comparison to my usual portion size, this

portion is….”. The estimation of the portion sizes was made using a 100mm-visual analog scale (VAS). For each picture, the participants were asked to adjust the VAS scale depending on their portion size preferences. The starting settings placed the indicator in the middle of the scale indicating “the picture shown represents my preferred portion size”. The extremes of the scale were labeled much smaller and much larger respectively. If the portion size was estimated as too big the participants were asked to shift the indicator proportionally to the right and if too small, to the left. For each participant, the picture for which the closes portion size to the ideal (the closes to the middle of the VAS scale) was chosen was selected and the value (gram) of the food was used for the analysis. In the rare cases where two pictures obtained exactly the same estimation, and mean of two portions was chosen for the analysis.

Since some of the dishes in this survey contained either meat or fish, it was important to identify the participant’s diet limitations and potential aversion towards certain dishes. Therefore, if the individual followed a diet other than omnivore or flexitarian, they were encouraged to estimate portion sizes only where applicable in reference to their preferred diet form. Participants stating to be vegans were thanked for their time, whereupon the questionnaire ended.

In order to facilitate the participation and to increase the number of participants, there were no specifications or limitations of the screen size used by the participants to fill the survey.

In order to exclude potential bots completing the surveys, the questionnaires were pre-tested for the estimated time needed to fill the survey. If the registered time was too short and unrealistic (consistently less than 2 seconds per picture) the data would be excluded. However, this was not the case for any of the participants.

Adult (over the age of 16) Austrian participants who completed the questionnaire entirely were included. Incomplete questionnaires were not included in the analysis. Besides incomplete answers, misinterpreting the VAS and unclear statements also lead to the exclusion of the data.

2.3. Assessment of eating behavior

To assess the eating habits of participants, the German version of the Dutch Eating Behavior Questionnaire (DEBQ) based on Grunert was used in the FEV-I form/Typ [3]. This specific questionnaire encompasses 33 questions in three categories [4]. The Dutch Eating Behavior Questionnaire (DEBQ) is designed to examine the dietary behavior of participants in three categories: restrictive, emotional, and externally determined (susceptibility to cues). All questions are answered using a 5-Point-Likert-Scale: “never”, “hardly ever”, “sometimes”, “often”, “very often”; corresponding to scale 1-5.

Studies design was approved by the ethics committee of the University of Vienna (approval ID 00576). All participants gave their written consent to participate in the studies.

Supplementary tables

Supplementary table 1. Descriptive statistics of study I. The study participants were grouped in BMI. gender. age. and hunger categories. The percentage of population was calculated in proportion to gander. age. and hunger groups.

		**Total**		**BMI <18.5**		**BMI 18.5-25**		**BMI 25-30**		**BMI>30**	
		**N**	**%**	**N**	**%**	**N**	**%**	**N**	**%**	**N**	**%**
**Gender**	Female	228	52.29	13	5.70	92	40.35	62	27.19	61	26.75
	Male	208	47.71	28	13.46	110	52.88	54	25.96	16	7.69
**Age**	16-25	126	29	24	19.05	64	50.79	26	20.63	12	9.52
	25-35	144	33	13	9.03	66	45.83	40	27.78	25	17.36
	35-45	81	19	3	3.70	40	49.38	14	17.28	24	29.63
	45-55	62	14	1	1.61	24	38.71	24	38.71	13	20.97
	>55	23	5	0	0.00	8	34.78	12	52.17	3	13.04
**Hunger**	Full	193	44.27	6	10.36	81	41.97	51	26.42	41	21.24
	Somewhat hungry	62	14.22	3	4.84	29	46.77	20	32.26	10	16.13
	Moderately hungry	108	24.77	12	11.11	60	55.56	22	20.37	14	12.96
	Very hungry	73	16.74	6	8.22	32	43.84	23	31.51	12	16.44
**Age (years)**	Mean± SD	33.96±11		26.29±7		32.75±11		37.02±13		36.61±10	
**High (cm)**	Mean± SD	173.18±9		180.76±9		174.24±9		172.54±8		167.34±8	
**Weight (kg)**	Mean± SD	75.25±17		56.02±6		66.40±9		81.40±9		99.44±16	
**BMI (kg/m^2)**	Mean± SD	25.26±6		17.13±1		21.83±2		27.29±1		35.52±5	

Supplementary table 2. Comparison of the mean values and % differences of the estimated weight data with the actual weight values for categories age and hunger.

	Age groups							Hunger					
**Item**	**16-25**	**25-35**	**35-45**	**45-55**	**>55**	**F**	**p**	**Full (%)**	**Somewhat**	**Moderately**	**Very hungry**	**F**	**p**
**Potatoes**	-2.80	6.92	3.52	7.80	2.07	2.114	0.078	5.1	-2.5	3.8	3.1	1.212	0.305
**Potato chips**	191.63	175.24	202.96	191.45	69.57	1.740	0.140	202.8	237.2	168.8	126.6	3.017	**0.030**
**Cake**	22.88	29.00	23.75	36.48	19.33	0.815	0.516	29.1	22.0	19.2	29.1	1.240	0.295
**Beans**	62.69	78.13	75.38	78.80	47.30	0.386	0.831	72.5	70.8	64.5	80.0	0.076	0.973
**Paprika**	7.66	15.36	19.26	6.41	-17.50	3.302	**0.011**	14.5	14.9	-0.5	11.3	0.695	0.555
**Apple compote**	-51.02	-49.76	-47.80	-49.66	-56.80	0.490	0.743	-49.9	-53.0	-54.2	-45.6	2.006	0.112
**Berries**	-30.42	-26.48	-12.58	-20.46	-28.30	3.089	**0.016**	-22.4	-28.4	-31.3	-21.9	0.808	0.490
**Noodles**	98.14	95.71	103.49	96.97	63.94	0.413	0.800	100.6	97.2	89.7	93.7	0.136	0.938
**Rice**	65.24	76.27	81.48	61.69	45.65	1.964	0.099	73.3	60.2	61.9	71.6	2.114	0.098
**Pork meat**	61.80	63.28	73.65	66.41	44.34	0.704	0.589	70.0	52.5	56.1	52.6	0.613	0.607
**Ham**	-6.95	-14.46	-11.09	-8.49	-12.95	0.851	0.494	-10.2	-13.4	-5.9	-18.7	0.758	0.518
**Fish fillet**	3.53	3.37	11.45	-0.68	4.44	2.209	0.067	8.1	0.0	-2.7	3.6	0.547	0.650
**Butter**	88.57	165.83	53.46	76.94	117.83	2.210	0.067	96.4	150.5	133.5	138.6	2.014	0.111
**Scrambled eggs**	-20.76	-9.43	-8.11	-5.65	-9.09	1.532	0.192	-10.2	-3.9	-14.9	-10.7	0.758	0.518

Supplementary table 3. Study II descriptive statistics. The study participants were grouped based on BMI. gender. age. education. physical activity. and hunger categories. The percentage of the population was calculated in proportion to gander. age. and hunger groups categories.

		Total		BMI <18.8		BMI 18.5-25		BMI 25-30		BMI>30	
		N	%	N	%	N	%	N	%	N	%
**Gender**	Female	380	52.41	14	3.68	208	54.74	90	23.68	68	17.89
	Male	354	47.59	6	1.74	177	51.30	111	31.17	51	14.78
**Age**	16-25	209	28.83	13	6.22	143	68.42	43	20.57	10	4.78
	25-35	231	31.68	5	2.16	125	54.11	71	30.73	30	12.99
	35-45	152	20.97	1	0.65	65	42.76	39	25.65	47	30.92
	45-55	91	12.55	1	1.10	37	40.66	30	32.96	23	25.27
	>55	42	5.79	0	0.00	16	38.10	17	40.47	9	21.43
**Hunger**	Full	401	57.78	8	2.00	210	52.37	107	26.68	76	18.95
	Somewhat hungry	162	20.78	6	3.70	88	54.32	45	27.78	23	14.20
	Moderately hungry	112	15.45	5	4.46	55	49.11	37	33.04	15	13.39
	Very hungry	50	5.98	1	2.00	33	66.00	11	22.00	5	10.00
**Age (years)**	Mean± SD	34.14±12		25.90±9		32.07±12		36.20±12		39.69±11	
**High (cm)**	Mean± SD	174.21±9		171.45±12		174.06±9		174.44±9		174.25±9	
**Weight (kg)**	Mean± SD	77.94±19		52.30±7		67.10±10		82.57±10		108.19±18	
**BMI (kg/m^2)**	Mean± SD	25.58±6		17.74±1		22.10±2		27.14±1		35.60±5	

Supplementary table 4. Estimations of perfect portion size in different BMI categories expressed as difference of the % change compared to the estimation of the whole population.

**Meal**		**BMI**				**F**	**P**
	**Average estimation**	**<18.5**	**18.5-25**	**25-30**	**>30**		
Spaghetti	253.94±97	2.78	-1.53	0.80	2.72	3.363	**0.018**
Chicken	166.40±74	-12.00	-2.67	1.48	8.08	3.903	**0.009**
Salmon	158.31±76	-11.57	-0.21	-2.70	6.90	3.917	**0.009**
Schnitzel	128.22±46	-16.16	-0.13	0.50	1.59	9.638	**<0.001**
Pizza	329.46±90	-6.59	-1.68	0.95	5.20	5.177	**0.002**
Goulash	297.30±107	-10.87	-1.41	2.17	2.46	1.539	0.203
Rice	368.16±98	-3.58	-0.04	-1.54	3.28	3.056	**0.028**
French fries	160.05±61	-1.59	-0.29	-0.98	-0.51	6.450	**<0.001**
Kaiserschmarrn	205.37±54	7.13	0.49	-2.74	1.93	1.273	0.282
Fruit salad	202.27±73	1.23	0.61	2.74	-6.65	1.396	0.243
Ice cream	76.89±34	-4.41	-2.11	2.22	3.50	6.052	**<0.001**
**Average**		-5.06	-0.79	-0.44	2.60		

Supplementary table 5. Estimations of perfect portion size in different age categories. The data represents ideal portion estimations in gram and in % change compared to the estimations of the whole population.

	**Age groups**											
	**16-25**		**25-35**		**35-45**		**45-55**		**>55**			
**Meal**	**Gram±SD**	**%**	**Gram±SD**	**%**	**Gram±SD**	**%**	**Gram±SD**	**%**	**Gram±SD**	**%**	**F**	**p**
Spaghetti	271.44±99	6.89	246.42±92	-2.96	245.53±99	-3.31	253.85±101	-0.04	238.57±94	-6.05	4.205	**0.002**
Chicken	171.17±75	2.87	166.88±72	0.29	160.12±73	-3.77	168.96±75	1.54	157.14±79	-5.56	1.948	0.101
Salmon	164.90±80	4.17	161.23±78	1.85	146.33±63	-7.57	160.67±78	1.49	147.62±77	-6.75	2.263	0.063
Schnitzel	136.12±51	6.17	126.84±43	-1.07	122.04±41	-4.82	127.53±51	-0.54	120.24±37	-6.22	3.053	**0.016**
Pizza	335.74±94	1.91	333.67±86	1.28	327.92±83	-0.47	305.68±97	-7.22	332.29±90	0.86	2.360	0.052
Goulash	307.18±101	3.32	302.17±113	1.64	276.64±98	-6.95	299.44±118	0.72	291.67±100	-1.90	3.903	**0.004**
Rice	377.99±97	2.67	366.52±102	-0.45	367.11±100	-0.29	353.30±87	-4.04	364.29±103	-1.05	7.234	**<0.001**
French fries	167.23±61	4.49	157.95±62	-1.31	154.14±58	-3.69	159.17±66	-0.55	159.52±63	-0.33	2.895	**0.021**
Kaiserschmarrn	212.22±53	3.34	203.30±55	-1.00	198.21±52	-3.48	207.58±57	1.08	203.81±57	-0.76	7.11	**<0.001**
Fruit salad	211.93±76	4.78	196.59±69	-2.81	203.58±71	0.65	197.38±74	-2.41	191.33±71	-5.41	2.603	**0.035**
Ice cream	77.16±35	0.35	77.76±34	1.13	75.39±43	-1.95	77.14±37	0.32	75.71±32	-1.53	0.675	0.609
**Average**		3.72		-0.31		-3.24		-0.88		-3.16		

Supplementary table 6. Statistical analysis of the impact of hunger level on ideal portion estimation. The analysis for data sets was performed by applying one-way ANOVA.

	Hunger									
	Full		Somewhat hungry		Moderately hungry		Very hungry			
**Meal**	**Gram±SD**	**%**	**Gram±SD**	**%**	**Gram±SD**	**%**	**Gram±SD**	**%**	**F**	**p**
Spaghetti	246.83 ±96	-2.80	254.63 ±97	0.27	260.00	2.39	293.75	15.68	3.670	**0.012**
Chicken	160.47 ±73	-3.53	168.06 ±71	1.03	177.79	6.88	182.81 ±78	9.90	1.334	0.262
Salmon	154.57 ±73	-2.36	155.56 ±72	-1.74	164.41	3.86	185.11 ±81	16.93	2.655	**0.048**
Schnitzel	125.44 ±44	-2.17	127.47 ±45	-0.58	130.80	2.02	147.92 ±62	15.37	3.463	**0.016**
Pizza	321.60 ±91	-2.39	332.48 ±93	0.92	344.96	4.71	344.21 ±92	4.48	2.172	0.090
Goulash	292.23 ±108	-1.71	286.11	-3.76	311.61	4.81	343.75	15.62	4.420	**0.004**
Rice	364.96 ±100	-0.87	362.65 ±94	-1.50	381.53	3.63	381.25 ±94	3.55	1.324	0.265
French fries	156.94 ±62	-1.94	158.39 ±59	-1.04	167.57	4.70	171.88 ±63	7.39	2.356	0.071
Kaiserschmarrn	204.74 ±55	-0.31	199.63 ±52	-2.79	208.02	1.29	221.49 ±51	7.85	2.504	0.871
Fruit salad	197.72 ±71	-2.25	202.39 ±76	0.06	203.13	0.43	236.31 ±72	16.83	1.355	0.256
Ice cream	75.38 ±34	-1.97	76.02 ±34	-1.13	78.11 ±32	1.58	89.36 ±38	16.22	3.028	**0.039**
Average		-2.03		-0.93		3.30		11.80		

1. Rust, P.; Hasenegger, V.; König, J., Österreichischer Ernährungsberich 2017. **2017**.
2. Almiron-Roig, E.; Solis-Trapala, I.; Dodd, J.; Jebb, S. A., Estimating food portions. Influence of unit number, meal type and energy density. *Appetite* **2013,** *71*, 95-103.
3. Grunert, S. C., Ein Inventar zur Erfassung von Selbstaussagen zum Ernährungsverhalten [An inventory for determination of eating behaviors through self- reporting]. *Diagnostica 35* (2), 167-179.
4. Van Strien, T.; Frijters, J. E.; Bergers, G.; Defares, P. B., The Dutch Eating Behavior Questionnaire (DEBQ) for assessment of restrained, emotional, and external eating behavior. . *International Journal of Eating Disorders* **1986,** *5*, 295–315.
